# Molecular‐Scale Tuning of Low‐Molecular‐Weight Gelators Controls Supramolecular Assembly and Directs Human Mesenchymal Stem Cell Growth

**DOI:** 10.1002/anie.202523454

**Published:** 2026-01-24

**Authors:** Chayanan Tangsombun, Amy Simpson, Rebecca L. Charlton, Lucy E. Sabin, Paul G. Genever, David K. Smith

**Affiliations:** ^1^ Department of Chemistry University of York Heslington York YO10 5DD UK; ^2^ Department of Biology University of York Heslington York YO10 5DD UK

**Keywords:** Gel, Multi‐component, Self‐assembly, Stem cells, Supramolecular

## Abstract

We report the synthesis and characterization of a simple alcohol‐functionalized low‐molecular‐weight gelator (LMWG) based on a 1,3:2,4‐dibenzylidenesorbitol scaffold (DBS‐CH_2_OH) and explore its self‐assembly in comparison to acylhydrazide‐functionalized DBS‐CONHNH_2_. DBS‐CH_2_OH forms a stiffer, highly organized, less‐soluble self‐assembled hydrogel, with a different assembly mode and much higher entropy/enthalpy of dissociation. On mixing, the LMWGs co‐assemble into gel nanofibers with similar thermodynamics to DBS‐CONHNH_2_. DBS‐CH_2_OH is an excellent scaffold for the growth of human mesenchymal stem cells (hMSCs), which exhibit extended spread‐shaped morphologies on the gel surface. In contrast, for DBS‐CONHNH_2_, hMSCs have a rounded morphology and penetrate into the softer gel. A simple functional group change on the LMWG, therefore, leads to a remarkable change in cell growth outcomes. When hMSCs are grown on the co‐assembled gel, they have the rounded morphology characteristic of DBS‐CONHNH_2_ but remain on the gel surface, like DBS‐CH_2_OH. The multi‐component gel thus shares characteristics of the individual LMWGs, with hMSC growth being controlled by factors like structure, stiffness, and dynamics. This work demonstrates how chemical manipulation of gels based on deceptively simple LMWGs can have profound impacts in biomaterials engineering.

## Graphical abstract

**Chemistry in charge of biology!** Simple low‐molecular‐weight gelators, with minor modifications in molecular structure, self‐assemble differently and can also co‐assemble when simultaneously triggered. This creates a family of gels with different properties that direct the growth of stem cells in different ways.

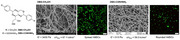

## Introduction

Supramolecular hydrogels based on the self‐assembly of low‐molecular‐weight gelators (LMWGs) are versatile soft materials with wide‐ranging applications.^[^ ^1^ ^]^ One area of particular interest is their use in tissue engineering, where their resemblance to extracellular matrix means some of them support and direct cell growth.^[^ ^2^, ^3^ ^]^ LMWG hydrogels can offer advantages over more commonly used polymer hydrogel scaffolds,^[^ ^4^ ^]^ such as their molecular‐scale tunability, reversibility, and adaptiveness. Although LMWG self‐assembly is increasingly well understood, it remains difficult to design new gelators, and in many cases, they are discovered by modifying well‐established systems. Another way of expanding their scope is to combine multiple LMWGs into integrated multi‐component gels.^[^ ^5^, ^6^, ^7^, ^8^ ^]^ On mixing LMWGs, there are a variety of possible outcomes; they may: (i) disrupt one another's assembly; (ii) preferentially interact with each other, forming a co‐assembled structure; (iii) preferentially interact with themselves, leading to self‐sorting.^[^ ^9^, ^10^, ^11^, ^12^, ^13^, ^14^, ^15^, ^16^ ^]^ As such, a multi‐component approach can tune and enhance performance, potentially extending LMWG applications.

In tissue engineering, it remains challenging to use supramolecular hydrogels to direct the growth of human stem cells, as they are relatively sensitive and difficult to grow in comparison to the more robust cancer cell lines or fibroblasts that are frequently studied with LMWG materials. A number of LMWGs capable of supporting stem cell growth have been reported, with most success being achieved using peptide LMWGs, which have highly tunable structures that can impact stem cell growth outcomes.^[^ ^17^, ^18^, ^19^, ^20^, ^21^, ^22^, ^23^, ^24^, ^25^ ^]^ For example, active signalling peptides, such as RGD or IKVAV, can be incorporated to encourage cell adhesion and spreading.^[^ ^26^, ^27^ ^]^ This can also be achieved by co‐assembling modified peptide fragments into a peptide hydrogel.^[^ ^28^, ^29^ ^]^ There are a variety of ways to influence outcomes in terms of stem cell growth—for example, gel stiffness can play an active role in differentiation.^[^ ^30^, ^31^, ^32^, ^33^, ^34^ ^]^ Furthermore, there has been considerable recent interest in the impact of LMWG hydrogel dynamics on cell growth.^[^ ^35^, ^36^, ^37^, ^38^, ^39^ ^]^ Multi‐component approaches, in which several LMWGs are mixed, are also a powerful way of tuning supramolecular stem cell growth scaffolds.^[^ ^40^, ^41^, ^42^, ^43^ ^]^

**Figure 1:**
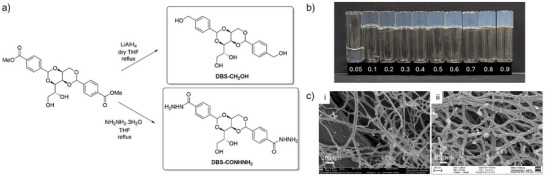
a) Synthesis of DBS‐CH_2_OH and DBS‐CONHNH_2_ from DBS‐CO_2_Me. b) Photographs of gels formed by DBS‐CH_2_OH at different loadings (%wt/vol) in H_2_O. c) SEM images of i) DBS‐CH_2_OH and ii) DBS‐CONHNH_2_, indicating the self‐assembly of nanoscale fibrillar networks in each case, scale bars 200 nm.

In contrast to peptide hydrogels, LMWGs based on sugar scaffolds have been less widely explored, perhaps surprisingly given the dominance of sugar‐based polymeric hydrogels in biomaterials engineering.^[^ ^44^ ^]^ Fitremann and co‐workers recently pioneered supramolecular gels based on alkylgalactonamides for stem cell growth.^[^ ^45^, ^46^, ^47^ ^]^ Sugars have also been incorporated into peptide gels.^[^ ^48^, ^49^, ^50^ ^]^ We have been particularly interested in sugar‐derived hydrogels based on 1,3:2,4‐dibenzylidenesorbitol (DBS).^[^ ^51^ ^]^ This synthetically accessible scaffold is commercially relevant, being widely used in industry, and can be modified on the “wingtips” to induce hydrogelation. We have used DBS‐CONHNH_2_ (Figure 1) as a cytocompatible scaffold for human mesenchymal stem cells (hMSCs).^[^ ^52^, ^53^, ^54^, ^55^ ^]^

As a result of our previous work, we became interested in the ability of other DBS derivatives to support hMSCs. As noted in an outstanding recent review by Dankers and co‐workers,^[^ ^56^ ^]^ there is an urgent need to develop simple rules to understand how chemistry can recreate the extracellular matrix. Although some principles have emerged (see above), the field remains a long way from a simple ‘Lipinski Rule of 5′ type understanding of LMWGs. As such, fundamental studies of LMWGs as stem cell growth scaffolds are of key importance, with a particular focus on gaining greater control over the proliferation and differentiation of such cells via the unique 3D‐environments that can be provided by these materials. Furthermore, many LMWGs used for cell growth are structurally complex—simple LMWGs are of high value, and probing the response of stem cells to small structural changes in such LMWGs is critical in enhancing understanding of such materials and creating a portfolio of new materials capable of intervening in stem cell biology.

We therefore targeted the synthesis of DBS‐CH_2_OH (Figure 1). We believed that, like DBS‐CONHNH_2_, it may be useful as a scaffold for hMSCs, and that studies would enhance the structure‐activity relationship understanding of such gels. Furthermore, multi‐component systems combining both LMWGs may introduce tunability and help understand performance. Herein, we report DBS‐CH_2_OH as a hydrogelator for the first time, explore its co‐assembly with DBS‐CONHNH_2_, and discover, to our surprise, that the two LMWGs induce very different outcomes in hMSC culture.

## Results and Discussion

### Synthesis and Characterization of DBS‐CH_2_OH

The synthesis of DBS‐CH_2_OH was previously reported by Nagarajan and co‐workers, who explored its ability to form gels in a variety of polar protic organic solvents and deep eutectics.^[^ ^57^ ^]^ We attempted to reproduce their synthesis, in which terephthalaldehyde was mono‐reduced using NaBH_4_ to 4‐hydroxymethylbenzaldehyde, and then reacted with d‐sorbitol to form DBS‐CH_2_OH. However, in our hands, the second step was unsuccessful. We therefore attempted an alternative two‐step method (Figure 1a) via our well‐established compound DBS‐CO_2_Me, synthesized by reaction of 4‐carboxybenzaldehyde methyl ester with d‐sorbitol in the presence of *p*‐TsOH.^[^ ^58^, ^59^, ^60^ ^]^ The resulting diester was reduced using an excess of 1 M LiAlH_4_ in dry THF under reflux conditions. After work‐up, we obtained pure DBS‐CH_2_OH in a yield of 68%. DBS‐CONHNH_2_ was also synthesized from DBS‐CO_2_Me (Figure 1a) using our previously reported methodology.^[^ ^58^ ^]^

The ^1^H NMR spectrum of DBS‐CH_2_OH showed new signals for the benzylic alcohol O‐H groups (δ 5.20 ppm) and CH_2_ groups (δ 4.50 ppm), while the methyl ester CH_3_ signal (δ 3.85 ppm) was no longer present (Figure S1). ^13^C NMR indicated the loss of the C═O group (Figures S2 and S3). ESI‐MS presented a peak [M + Na]^+^ at *m/z* value of 441.1515, corresponding to the appropriate calculated mass (Figure S4). In the FT‐IR spectrum, a strong broad band at 3205 cm^−1^ was assigned to the Ar‐CH_2_‐OH alcohol group and the hydroxyl groups of the d‐sorbitol backbone, while the band at 1724 cm^−1^ assigned to the C═O of DBS‐COOMe had disappeared. For full characterization data, see Supporting Information.

### Gel Characterization of DBS‐CH_2_OH and Comparison with DBS‐CONHNH_2_

In their previous work,^[^ ^57^ ^]^ Nagarajan and co‐workers reported DBS‐CH_2_OH as insoluble in water and unable to form hydrogels via a heat‐cool cycle. Our experience with DBS derivatives, however, led us to reason that it must be right on the edge of water‐solubility and gel assembly. We therefore first sonicated DBS‐CH_2_OH in water (1 mL) to obtain a fine suspension, then heated with a heat gun in a sealed vial until completely dissolved, and cooled under ambient conditions for 2 h. We were delighted to find this gave reproducible sample‐spanning hydrogels with a minimum gelation concentration (MGC) of just 0.15% wt/vol (Figure 1b). Above 0.40% wt/vol, DBS‐CH_2_OH became difficult to fully dissolve, and did not form completely homogeneous gels, but it still achieved vial inversion. Similar behavior is observed for DBS‐CONHNH_2_, which forms homogeneous gels in the range 0.28%–0.40% wt/vol. It is notable that DBS‐CH_2_OH exhibits a lower MGC value than DBS‐CONHNH_2_, which may reflect greater assembly potential (see below).

At the macroscopic level, *T*_gel_ values were determined using a tube inversion methodology (Table 1). At a loading of 0.20% wt/vol, DBS‐CH_2_OH had a *T*_gel_ value of 72 °C, which increased to 90 °C at a loading of 0.30% wt/vol. This is very similar to the *T*_gel_ value of DBS‐CONHNH_2_, which is 88 °C at 0.30% wt/vol. Rheometry was then performed using a parallel plate geometry on gels that had been formed in a  ″bottomless″ sample vial and then transferred to the rheometer plate at room temperature (Table 1). All hydrogels had Gʹ > Gʺ, with Gʹ being independent of frequency, indicative of gel formation (Figures S17–S19). The Gʹ value of DBS‐CH_2_OH was 1560 Pa at a loading of 0.20% wt/vol, rising to 3430 Pa at 0.30% wt/vol. This was significantly stiffer than the gels formed by DBS‐CONHNH_2_, which had a Gʹ value of 610 Pa at 0.30% wt/vol. None of these gels exhibit any swelling when exposed to excess solvent, and we did not observe significant differences in performance when performing rheology at 37 °C—therefore, we conclude that these studies directly reflect the rheological properties of gels used later in this report for hMSC growth. We also tested the hydrophilicity of the gels via contact angle measurement—in all cases, the water droplet immediately spread over and into the surface of the gel, preventing an actual contact angle measurement, and indicating that, as expected, these are very hydrophilic materials.

**Table 1:** Macroscopic characterization of gels based on DBS‐CH_2_OH and DBS‐CONHNH_2_. *T*_gel_ values determined by inverted vial methods, rheological data obtained from rheometry using a parallel plate geometry (*N* = 3, mean reported).

LMWG	Loading/% wt/vol	*T*_gel_/°C	Gʹ/Pa	Gʺ/Pa	Tan δ	Gʹ = Gʺ/% strain
DBS‐CH_2_OH	0.20%	72	1560	120	0.077	12.6
DBS‐CH_2_OH	0.30%	90	3430	270	0.079	14.3
DBS‐CONHNH_2_	0.30%	88	610	50	0.082	12.6

For nanoscale characterization, we made use of TEM and SEM. We note that for TEM/SEM imaging on gels, there is a risk of drying artefacts—we do not use the imaging here to provide analysis of (e.g.,) porosity, but rather as a simple technique to compare the observable assembled nanoscale morphologies of gel samples that have been treated in an identical way. In this regard, TEM/SEM can sometimes provide useful comparative insights. TEM samples were produced by mobilizing the gel using a whirlimixer for ca. 10 s and pipetting 3 µl onto a TEM grid, allowing it to adsorb for 60 s and wicking off excess material before negative staining. TEM images of DBS‐CH_2_OH showed long fibers with diameters of 15–35 nm (Figures S13 and S14), while DBS‐CONHNH_2_ exhibited a twisted nanostructure with a width of 25–45 nm (Figures S13 and S15). It was therefore possible to distinguish between morphologies. Gel samples were prepared for SEM by depositing a thin smear of gel on a small copper shim, followed by plunge freezing in slushy nitrogen and freeze‐drying. SEM demonstrated the presence of an entangled nanofiber network, but it was not possible to unambiguously identify differences between LMWGs (Figures 1c, S10, S11, and S13).

On the molecular‐scale, ^1^H NMR is a powerful technique for understanding gel assembly as it only detects mobile, liquid‐like molecules in a gel, and hence, with the use of a mobile internal standard, can quantify both mobile, liquid‐like and assembled, solid‐like molecules.^[^ ^61^ ^] 1^H NMR studies were initially performed at room temperature to confirm the assembly of gelator molecules (Figure S5). DBS‐CH_2_OH gels were prepared in D_2_O at a loading of 0.3% wt/vol via a sonication‐heat‐cool cycle, with the addition of DMSO (1.4 µL) as an internal standard. After standing for 24 h, a ^1^H NMR spectrum was recorded at 25 °C, indicating >98% assembly. The same was observed for DBS‐CONHNH_2_.

**Figure 2:**
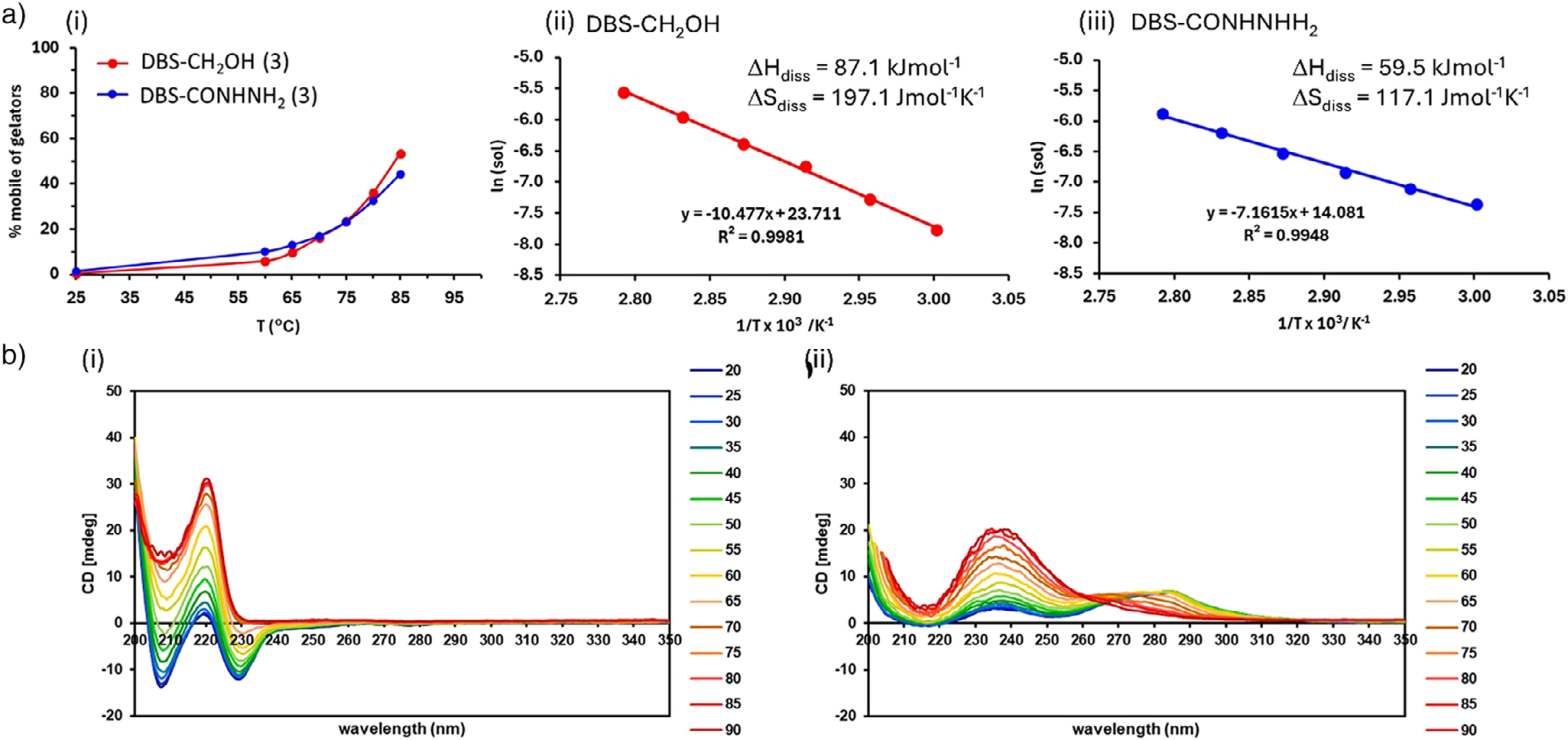
a) Data from VT ^1^H NMR experiments performed on hydrogels of DBS‐CH_2_OH and DBS‐CONHNH_2_ (0.30% wt/vol loading). i) Plot of gelator mobility (%) against temperature for DBS‐CH_2_OH (red) and DBS‐CONHNH_2_ (blue) indicating the greater temperature sensitivity of DBS‐CH_2_OH. ii) Van't Hoff plot and thermodynamic parameters derived for DBS‐CH_2_OH. iii) Van't Hoff plot and thermodynamic parameters derived for DBS‐CONHNH_2_. b) VT CD data indicating the change in spectrum from room temperature (blue) to 90 °C (red) in steps of 5 °C, for i) DBS‐CH_2_OH and ii) DBS‐CONHNH_2_.

The samples were then studied at increasing temperatures to understand how the gel responds. Increasing temperature results in the disassembly and dissolution of gelator molecules, which then appear in the ^1^H NMR spectra (Figures 2a‐i, S6, and S7). On heating, the LMWG peaks for Ar‐H and Ar‐CH shift downfield, reflecting the fact that, in the assembled state, the hydrophobic effect plays a significant role in holding this gel together. Quantifying the amount of LMWG that can be observed in the spectrum allows us to conclude that by 85 °C, around 53% of DBS‐CH_2_OH became mobile, which reflects the *T*_gel_ value of this system (see above). For DBS‐CONHNH_2_, 44% of mobile LMWG was observed under the same conditions. Detailed measurements of LMWG solubility at different temperatures were then used to create van't Hoff plots (Equation 1) to calculate the thermodynamic parameters associated with the gel‐sol transition (Figure 2a).^[^ ^62^ ^]^ This assumes that the gel‐sol transition can be treated like a crystal‐solution transition, and provides a valuable quantitative thermodynamic insight into gel assembly.

(1) $$\ln\left[\mathrm{sol}\right]=\frac{H_{diss}}{RT}+\frac{S_{diss}}{R}$$

For DBS‐CH_2_OH (Figure 2a‐ii), LMWG solubility showed a much greater temperature sensitivity, with a large entropy of dissolution (Δ*S*_diss_) of 197 J mol^−1^K^−1^, and the enthalpy of dissolution (Δ*H*_diss_) being 87.1 kJ mol^−1.^ The positive entropy reflects the increase in disorder as the assembled gel is converted into a sol, while the large positive enthalpy reflects the energy required to break down the well‐organized non‐covalent interactions during this transition.

Surprisingly, although DBS‐CONHNH_2_ has a very similar *T*_gel_ value to DBS‐CH_2_OH, the thermodynamic detail of its solubility is very different, with a much shallower temperature sensitivity (Figure 2a‐i). For DBS‐CONHNH_2_ (Figure 2a‐iii), Δ*S*_diss_ was only 117 J mol^−1^K^−1^ and Δ*H*_diss_ had fallen to just 59.5 kJ mol^−1.^ This indicates that the self‐assembled structure of DBS‐CH_2_OH is significantly more organized than that of DBS‐CONHNH_2_, hence generating more disorder on disassembly, albeit at a greater enthalpic cost of breaking non‐covalent interactions. For such structurally similar gelators, we were surprised by how large this difference was, indicating that relatively small changes in the peripheral group can drive major changes in assembly thermodynamics. It suggests that the acyl hydrazide group in DBS‐CONHNH_2_ may somewhat hinder the organization of the rest of the DBS scaffold. The enthalpy‐entropy compensation commonly seen in supramolecular systems,^[^ ^63^, ^64^, ^65^ ^]^ and observed here, means both systems end up having similar *T*_gel_ values. However, at 37 °C, there are significant differences in solubility, with ln(Sol) values for DBS‐CH_2_OH and DBS‐CONHNH_2_ being calculated as −10.09 and −9.02, respectively, indicating solution‐phase concentrations of 41 and 120 µM (0.6% and 1.7% of the total) respectively DBS‐CH_2_OH is therefore significantly less soluble, and less dynamic at the molecular scale, than DBS‐CONHNH_2_ at cell growth temperature.

Circular dichroism (CD) spectra for DBS‐CH_2_OH (0.05% wt/vol) at room temperature displayed negative bands at ca. 230 and 208 nm, indicative of assembly of the sorbitol “bodies” of the LMWG (Figure 2b‐i). DBS‐CONHNH_2_ (0.05 wt/vol) had a positive band at ca. 275 nm (Figure 2b‐ii), which can be assigned to the π–π stacking of the aromatic wings.^[^ ^66^ ^]^ These longer wavelength CD bands were not observed for DBS‐CH_2_OH, confirming that the two similar LMWGs have very different assembly modes, possibly induced by differences in interactions between the aromatic “wings”. This is consistent with the very different thermodynamics of assembly for each LMWG.

Variable temperature CD spectroscopy, increasing the temperature from 20 to 90 °C, changed the intensity of the CD bands (Figure 2b). For DBS‐CH_2_OH (Figure 2b‐i), the negative bands became smaller while a new positive band at 220 nm increased. For DBS‐CONHNH_2_ (Figure 2b‐ii), the broad peak centered at 275 nm started to disappear, leading instead to a band centered at 235 nm. These changes reflect the transition from the self‐assembled forms of the two LMWGs to the solution phase CD spectra associated with the individual molecules.

### Multi‐Component Gel Characterization

We then moved on to consider multi‐component gels formed by combining DBS‐CH_2_OH with DBS‐CONHNH_2_. Pleasingly, gels were observed when using mixtures of these LMWGs and exposing them to a sonication‐heat‐cool cycle. Unlike previous multi‐component gels that incorporate DBS‐CONHNH_2_, in which stepwise self‐assembly has been used, resulting in self‐sorting,^[^ ^67^, ^68^ ^]^ here, both LMWGs are activated by the same trigger and should assemble simultaneously. We reasoned this may lead to a different outcome.

The *T*_gel_ value of a mixture of DBS‐CH_2_OH and DBS‐CONHNH_2_ (each 0.15% wt/vol) was 92 °C (Table 2). When combined at higher loadings (DBS‐CH_2_OH at 0.20% or 0.30% wt/vol and DBS‐CONHNH_2_ at 0.30% wt/vol), the *T*_gel_ value was >100 °C as the network became more extensive. Firstly, this indicated that these two LMWGs could assemble to form effective multicomponent hydrogels. Secondly, at total loadings of 0.30% wt/vol, the two individual LMWGs and the two‐component mixture all had similar *T*_gel_ values (88–92 °C). This is somewhat different from what is observed for some multi‐component systems, where the gel stability can be enhanced even at lower loadings as independent self‐sorted networks reinforce one another.^[^ ^67^, ^68^ ^]^ Once again, contact angle measurements indicated that these multi‐component gels were highly hydrophilic materials.

**Table 2:** Macroscopic characterization of multicomponent gels based on DBS‐CH_2_OH and DBS‐CONHNH_2_. *T*_gel_ values determined by inverted vial methods, rheological data obtained from rheometry using a parallel plate geometry (*N* = 3, mean reported).

DBS‐CH_2_OH/% wt/vol	DBS‐CONHNH_2_/% wt/vol	*T*_gel_ /°C	Gʹ/Pa	Gʺ/Pa	Tan δ	Gʹ = Gʺ/% strain
0.15%	0.15%	92	1915	95	0.050	10.1
0.20%	0.30%	>100	6210	345	0.056	5.3
0.30%	0.30%	>100	11230	700	0.062	5.0

Rheology indicated that the multi‐component hydrogel at an overall loading of 0.30% wt/vol (0.15% wt/vol each LMWG) had a Gʹ value of 1915 Pa (Table 2; Figures S20–S22), in between the Gʹ values of the two single‐component gels at 0.30% wt/vol (610 and 3430 Pa). Again, this may be suggestive of co‐assembly, where the network is somewhat intermediate between the two extremes. At higher LMWG loadings, the Gʹ value of the two‐component gel increased to 6200 and 11 233 Pa (Table 2). Combining the two LMWGs can avoid difficulties associated with the relatively low solubility of each individual LMWG to provide gels with total loadings up to 0.60% wt/vol that have significantly higher stiffness. At these higher loadings, the Gʹ/Gʺ crossover value decreased to ca. 5%, indicating that these more rigid gels were more easily fractured by application of shear.

To gain further insight into the rheological performance of the gels, we performed stress relaxation experiments. In each case, we rapidly applied a strain of 1.0% (within the LVR). We then monitored the dissipation of the internal stress built up within the material, giving an insight into the bulk dynamics of these materials. For each of the hydrogels based on DBS‐CONHNH_2_, DBS‐CH_2_OH, and the two‐component DBS‐CONHNH_2_/DBS‐CH_2_OH mixture, stress relaxation was very fast, taking a total of <1s (Figure S23). In each case, stress was very rapidly (ca. 0.1s) dissipated to a minimum, and then the gel slightly more slowly (ca. 1s) reorganized into its optimum structure. All gels showed similar relaxation profiles. Fast stress‐relaxing gels are considered to potentially facilitate cell spreading, proliferation, and differentiation as cells can mechanically remodel their surroundings—indeed, work on polymer gels suggests gels with faster stress relaxation exhibit enhanced stem cell proliferation and differentiation.^[^ ^69^ ^]^ However, the gels investigated here, which are based on the assembly of small molecules, dissipate stress much more rapidly than most previously studied polymeric gels.^[^ ^38^, ^69^ ^]^ One previous study on a supramolecular gel assembled from small polymeric components (ca. 20 kDa) reported stress relaxation rates that could be tuned (0.04–1300 s) based on structural modification.^[^ ^70^ ^]^ Our system shows a similar stress relaxation rate to the fastest of these systems, but we did not observe any tunability amongst our small molecules. In summary, therefore, although our NMR studies indicate a significant difference in molecular‐scale dynamics between gels, the network‐level bulk dynamics indicated by stress relaxation studies are all on a similar, very fast timescale.

TEM analysis of the two‐component system suggested the formation of a single nanostructure with a fiber diameter of 13–25 nm (Figures S13 and S16). We did not observe the larger bundles typically associated with DBS‐CONHNH_2_. This suggests the co‐assembly of the two LMWGs into a single integrated nanostructure rather than self‐sorted nanofibers. SEM data were supportive of this view (Figures S12 and S13).

**Figure 3:**
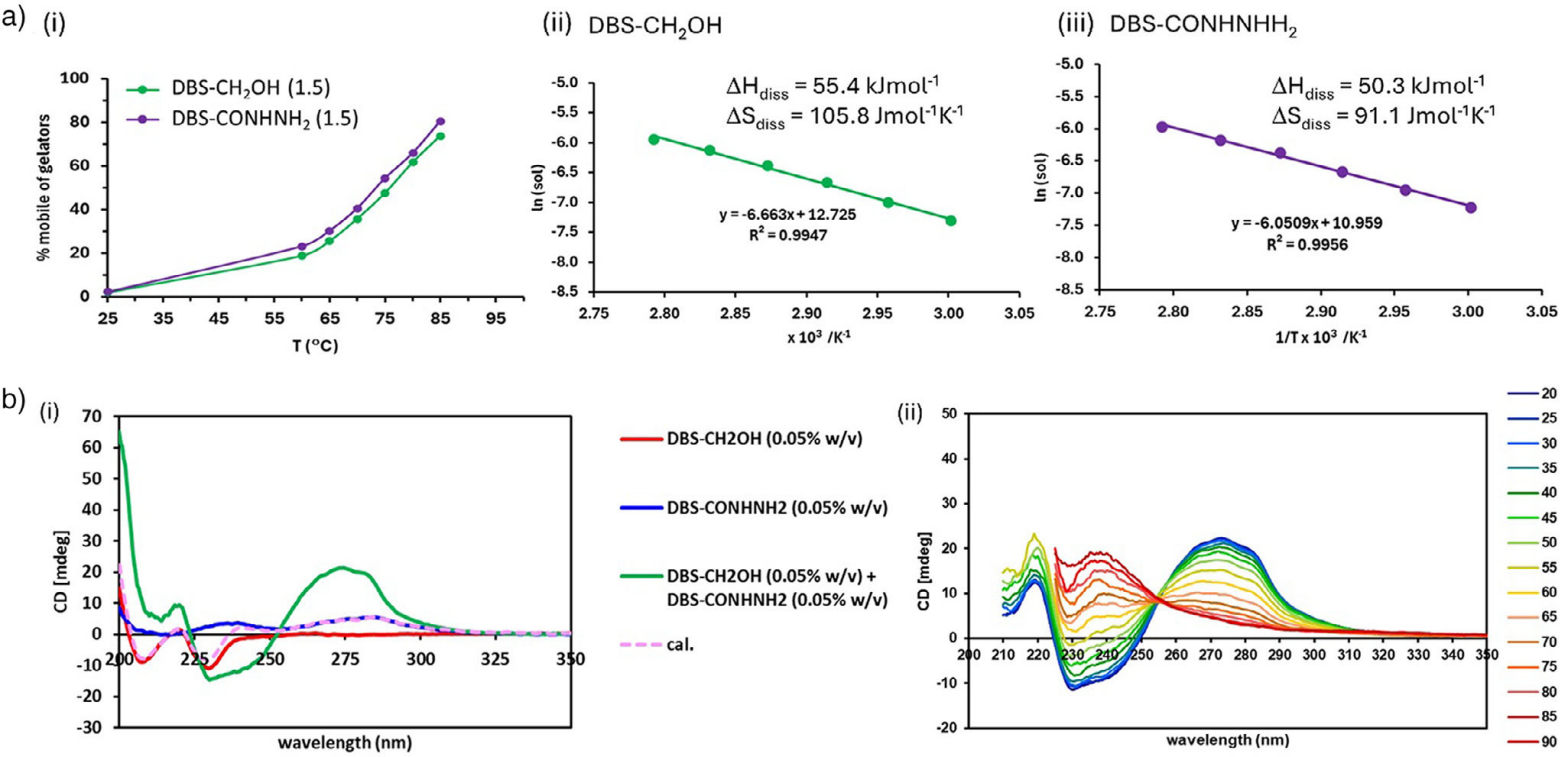
a) Data from VT ^1^H NMR experiments performed on a multi‐component hydrogel of DBS‐CH_2_OH and DBS‐CONHNH_2_ (0.30% wt/vol total loading, 0.15% wt/vol of each component). i) Plot of gelator mobility (%) against temperature for DBS‐CH_2_OH (green) and DBS‐CONHNH_2_ (purple). ii) Van't Hoff plot and thermodynamic parameters derived for DBS‐CH_2_OH in the multi‐component mixture. iii) Van't Hoff plot and thermodynamic parameters derived for DBS‐CONHNH_2_ in the multi‐component mixture. b) i) CD spectra of DBS‐CH_2_OH (0.05% wt/vol, red), DBS‐CONHNH_2_ (0.05% wt/vol, blue), DBS‐CH_2_OH + DBS‐CONHNH_2_ (each 0.05% wt/vol, green) and the calculated spectrum for a sum of DBS‐CH_2_OH and DBS‐CONHNH_2_ (dashed pink) demonstrating that the multi‐component gel (green) is different to the calculated sum of components (dashed pink). ii) VT CD data for the change in spectrum from room temperature (blue) to 90 °C (red) in steps of 5 °C, for the multi‐component gel formed by DBS‐CH_2_OH + DBS‐CONHNH_2_ (each 0.05% wt/vol).

We then performed VT ^1^H NMR on the multi‐component gel (0.15% wt/vol of each component). On increasing the temperature from 60 °C to 85 °C, signals from DBS‐CONHNH_2_ and DBS‐CH_2_OH were detected (Figures 3‐i and S8), the intensity of which increased with rising temperatures as the gel disassembled. Van't Hoff plots were created for each LMWG in the multi‐component system (Figure 3‐ii and iii), and we found that the entropy and enthalpy of dissolution were different from those for the individual LMWGs. For DBS‐CH_2_OH Δ*S*_diss_ was 106 J mol^−1^K^−1^ and Δ*H*_diss_ was 55.4 kJ mol^−1.^, while for DBS‐CONHNH_2_, Δ*S*_diss_ was 91.1 J mol^−1^K^−1^ and Δ*H*_diss_ was 50.3 kJ mol^−1.^ Both the enthalpy and entropy of disassembly were lower in magnitude than for either of the LMWGs individually. Indeed, DBS‐CH_2_OH had thermodynamic parameters that were very significantly lower. This suggests that the self‐assembled state in the multi‐component system is less well‐organized and forms weaker non‐covalent interactions than either individual gelator. Furthermore, the thermodynamic parameters measured for each LMWG in the multi‐component system were relatively similar, even though these LMWGs have very different thermodynamics when investigated individually. This supports their co‐assembly.

DBS‐CH_2_OH and DBS‐CONHNH_2_ in the multi‐component system are calculated to have ln(Sol) values of −8.77 and −8.56, respectively, at 37 °C, corresponding to concentrations in the solution phase of 155 and 192 µM (2.2% and 2.7% of the total LMWG), respectively. These observations suggest that the environment, interaction, and packing of LMWGs have changed, with co‐assembly of LMWGs into a combined structure with its own unique thermodynamic profile. The data suggest that DBS‐CONHNH_2_ is perhaps slightly less firmly held in the co‐assembled structure than DBS‐CH_2_OH, perhaps hinting at a more peripheral nature of DBS‐CONHNH_2_, although this difference is relatively small. Most importantly, this new co‐assembled structure is significantly less thermodynamically favored than DBS‐CH_2_OH and has five times higher solubility at cell growth temperatures. This would suggest that at the molecular level, the co‐assembled gel is significantly more dynamic than a gel based solely on DBS‐CH_2_OH.

CD spectrometry was also used to investigate the multi‐component system (at 0.05% wt/vol of each of DBS‐CH_2_OH and DBS‐CONHNH_2_). Broad positive and negative peaks were observed at 275 nm and 235–250 nm, respectively (Figure 3b‐i). The spectrum of the multi‐component system was very significantly different from the calculated sum of the two LMWGs individually. This would again support the view that rather than each LMWG assembling into its own self‐sorted nanostructure, they are co‐assembling into a new self‐assembled nanostructure, with its own emergent CD spectrum. On heating, the positive band at 275 nm decreased in intensity (Figure 3b‐ii), indicative of disassembly. The CD spectrum of the two‐component system recorded at 90 °C does then, at 90 °C, correspond to the sum of the two individual LMWGs (Figure S9), indicating that after disassembly, the system contains individual molecules of each LMWG which behave in an additive way. Overall, this proves that the multi‐component system forms a co‐assembled nanostructure with its own unique CD spectrum, which on heating disassembles into individual molecules that behave like the individual LMWGs.

### Biological Studies

**Figure 4:**
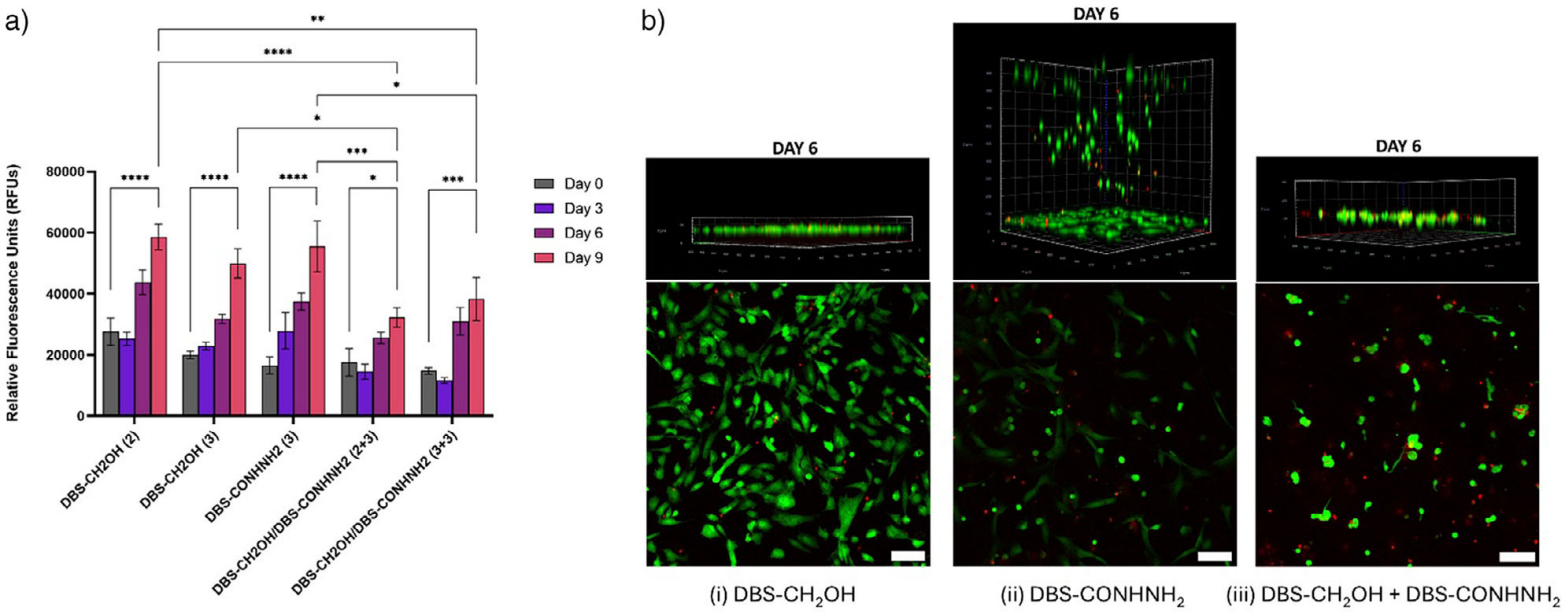
a) Metabolic activity of Y201 hMSCs cultured on DBS‐CH_2_OH (0.2% and 0.3% wt/vol), DBS‐CONHNH_2_ (0.3% wt/vol), and multi‐component DBS‐CH_2_OH/DBS‐CONHNH_2_ (0.2/0.3 and 0.3/0.3% wt/vol) gels measured using Alamar Blue assay. The number in brackets from left to right represents the concentration of DBS‐CH_2_OH and DBS‐CONHNH_2_ in mg mL^−1^, respectively. *n* = 6, mean reported with error bars represent standard error (mean ± SEM): ^*^ *p* < 0.05, ^**^ *p* < 0.01, ^***^ *p* < 0.001, ^****^ *p* < 0.0001 (two‐way ANOVA). b) 3D confocal microscopy images (top) and *z*‐axis maximum projection images (below) of Y201 hMSCs growth on i) DBS‐CH_2_OH (0.3% wt/vol), ii) DBS‐CONHNH_2_ (0.3% wt/vol) and iii) DBS‐CH_2_OH/DBS‐CONHNH_2_ (0.3/0.3% wt/vol) showing live stain (Calcein AM, green) and dead stain (PI, red) at day 6. Scale bar of 100 µm.

Having discovered that DBS‐CH_2_OH is a potent new LMWG and is also capable of co‐assembling with DBS‐CONHNH_2_, we moved on to performing biological studies of these systems. For this, we used an immortalized cell line (Y201), clonally isolated from a mixed population of human bone marrow stromal cells, which has been highly characterized to demonstrate hMSC characteristics.^[^ ^71^, ^72^ ^]^ Cytocompatibility of the different gels was evaluated by measuring the metabolic activity of Y201 hMSCs using the Alamar Blue assay.^[^ ^73^ ^]^ Hydrogel samples of DBS‐CH_2_OH (0.20% or 0.30% wt/vol), DBS‐CONHNH_2_ (0.30% wt/vol), and DBS‐CH_2_OH/DBS‐CONHNH_2_ (0.20% or 0.30% wt/vol for DBS‐CH_2_OH and 0.30% wt/vol for DBS‐CONHNH_2_) were fabricated in non‐treated 96‐well plates. Y201 hMSCs (25 000 cells/ well) were seeded and cultured on these gels over 9 days. The cells were metabolically active over the culturing time, demonstrating that all tested hydrogels are cytocompatible (Figure 4a). Pleasingly, there was no significant difference in metabolic activity between hMSCs cultured on DBS‐CH_2_OH and DBS‐CONHNH_2_. Given that DBS‐CONHNH_2_ has excellent cytocompatibility, in spite of having no specific cell adhesion motif,^[^ ^53^, ^54^, ^55^ ^]^ the discovery of DBS‐CH_2_OH as having equivalent potential for hMSC growth is an important finding. The cellular metabolic activity on the single‐component hydrogels was slightly greater than on multi‐component hydrogels—we suggest this may reflect the greater stiffness and different composition of the multi‐component system, which could impact cell adhesion, spreading, and penetration (see full discussion below). Nonetheless, all gels were effective scaffolds for hMSC growth.

Live‐dead staining was performed and imaged using confocal laser scanning microscopy to further assess the viability, morphology, and penetration of hMSCs within hydrogels. (Figure 4b). Calcein AM was used to stain live cells showing green fluorescence, while any dead cells, stained with propidium iodide, could be visualized via red fluorescence (Figure S30). Hydrogel samples (0.4 mL) were prepared in non‐treated 24‐well plates, followed by cell seeding (25 000 cells/well). In all cases, most cells remained alive, with very few dead cells observed. We quantified this observation for each of the gels and found that on DBS‐CONHNH_2_ and DBS‐CH_2_OH, >80% of cells were live at all timepoints (Figure S31). On the two‐component hydrogel, there were fewer cells, and the ratio of dead cells increased to ca. 40% by day 6—consistent with the slightly lower metabolic activity observed on these gels as described above. Overall, however, live‐dead staining supported the view that all hydrogels supported cell growth, in agreement with the Alamar blue test. Most interestingly, however, there was a significant difference in cell morphology when using different hydrogels. Y201 hMSCs cultured on DBS‐CH_2_OH showed an elongated spread morphology (Figures 4b, S25, and S26), whereas for DBS‐CONHNH_2_, a more rounded cell morphology was observed (Figures 4b and S27).

It is worth noting that hMSCs grown on plastic typically exhibit a spread, fibroblast‐like morphology, similar to that seen here on the DBS‐CH_2_OH. This is due to the cytoskeletal arrangement of the cells in response to the mechanical and/or chemical properties of the growth material. Furthermore, for the cells to have developed this spread morphology, there must be effective focal adhesion between the self‐assembled DBS‐CH_2_OH network and the hMSCs. The differences in cytoskeletal tension affect differentiation of hMSCs, with spread‐morphology cells (with higher tension) showing increased osteogenesis, while rounded cells are more commonly associated with adipogenesis.^[^ ^74^, ^75^, ^76^, ^77^ ^]^ We initially considered that the rounded cell morphology on the DBS‐CONHNH_2_ was due to the lower Gʹ value of this gel, while the stiffer DBS‐CH_2_OH may have encouraged cells to elongate (Table 3)—indeed, the response of hMSCs to this type of mechanical cue is well‐known.^[^ ^30^, ^31^, ^32^, ^33^, ^34^ ^]^ However, evidence from the multi‐component gel (see below) indicated that the system was not only responding to gel stiffness.

**Table 3:** Summary of key results in the paper highlighting differences between hydrogels and their cell growth potential.

DBS‐CH_2_OH	DBS‐CONHNH_2_	Gel Stiffness	Δ*H*_diss_ and Δ*S*_diss_	Solubility at 37 °C	Stress relaxation rate	Cell penetration	Cell shape
–	0.30%	Low	Medium	Medium	Very fast	Yes	Rounded
0.30%	–	Medium	High	Low	Very fast	No	Spread
0.30%	0.30%	High	Low	High	Very fast	No	Rounded

Considering hMSCs grown on the two different gels in more detail, it was also evident that the majority of the spread‐morphology cells grown on DBS‐CH_2_OH remained on the gel surface, while the rounded cells grown on DBS‐CONHNH_2_ penetrated into the gel, growing within the matrix and, having travelled through the gel, on the plastic at the bottom of the well plate. We hypothesized that the greater softness of DBS‐CONHNH_2_ might permit cell penetration (Table 3). Further, we initially speculated that penetration into the softer DBS‐CONHNH_2_ matrix might also be correlated with the rounded cell morphology, but studies of the multi‐component gel (see below) demonstrated that this simple hypothesis was incorrect.

To probe these effects in more detail, we cultured hMSCs on the multi‐component DBS‐CH_2_OH/DBS‐CONHNH_2_ gels (0.30% wt/vol of each component). In this case, the cells did not penetrate into the gel—this was expected because the stiffness of these gels is considerably higher than that of DBS‐CONHNH_2_ alone (Table 3; Figures S28 and S29). However, surprisingly to us, the cells grown on these very stiff multi‐component gels had a rounded morphology, similar to that seen on the much softer DBS‐CONHNH_2_. This was contrary to our initial expectation that the rounded cell shape seen on the DBS‐CONHNH_2_ was simply a response to stiffness‐associated mechanical cues and/or penetration into a soft 3D‐like environment. Indeed, the results summarized in Table 3 make clear that different factors must control cell penetration and cell shape in this system, as these outputs are not correlated with one another.

There are many factors that can affect cell morphology and penetration, such as nanofiber structure, pore size, macroscopic gel shape, mechanical properties, and chemical functionalization.^[^ ^78^, ^79^, ^80^, ^81^ ^]^ The results here suggest that cell penetration depends on gel stiffness, with cells only penetrating the softest gels based wholly on DBS‐CONHNH_2_. Penetration of cells into the gels is clearly not correlated with the rounded morphology, which therefore must originate from other factors. In terms of cell morphology, DBS‐CH_2_OH is the only gel in which cells with a spread morphology were observed. We suggest this is a result of more effective adhesion to the alcohol‐functionalized LMWG assemblies. Conversely, with gels that incorporate DBS‐CONHNH_2_, hMSCs favor a rounded growth morphology—even in the hybrid gel, this behavior persists. We suggest that the presence of DBS‐CONHNH_2_ is the trigger for the rounded cell morphologies, possibly as a result of its lack of adhesive interactions with hMSCs—even in the hybrid gel, the DBS‐CONHNH_2_ could mask the cell adhesive effect of DBS‐CH_2_OH. However, we also note that all of the gels containing DBS‐CONHNH_2_ were less well organized, with significantly lower dissociation enthalpies/entropies and higher solubilities at 37 °C, indicative of greater molecular‐scale dynamics (Table 3). This enhanced molecular‐scale mobility of gels containing DBS‐CONHNH_2_ may therefore also disrupt focal adhesions between the hMSCs and the self‐assembled nanofibers. We note, however, that on the bulk scale, the stress relaxation dynamics are the same for all gels, so bulk network dynamics cannot be impacting cell growth here.

## Conclusions

In summary, we report a new, commercially relevant, synthetically simple hydrogelator, DBS‐CH_2_OH, synthesized by reduction of DBS‐CO_2_Me using LiAlH_4_. This LMWG can be easily triggered to form gels via a sonication‐heat‐cool cycle with a low MGC (0.15% wt/vol). These gels are stiffer than gels formed by our well‐established DBS‐CONHNH_2_, have different nanoscale organization, much higher enthalpies and entropies of dissociation, and lower solubility at 37 °C. Multi‐component supramolecular hydrogels formed from the combination of DBS‐CH_2_OH and DBS‐CONHNH_2_ have a new integrated co‐assembled nanostructure with its own thermodynamic characteristics.

DBS‐CH_2_OH has excellent cytocompatibility and supports the growth of Y201 hMSCs, promoting them to adopt a spread morphology on the gel surface, suggesting strong focal adhesions between this new gel and hMSCs. This is in contrast to DBS‐CONHNH_2_‐containing gels, which promoted the cells to form a rounded shape. This is a remarkable case in which a simple change in functional group on an LMWG leads to very different outcomes in terms of the hMSC growth. All gels have similar, very fast, bulk stress relaxation dynamics, so this cannot be a factor in the different outcomes. In the softer DBS‐CONHNH_2_ gel, the rounded hMSCs penetrated into the gel, whereas in the stiffer DBS‐CH_2_OH, they remained on the surface. The two‐component systems combining both LMWGs also exhibited cell proliferation, giving rounded cells on the surface of the gel. Gel softness is the key factor allowing cell penetration, while the presence of DBS‐CONHNH_2_ plays a key role in the formation of rounded cells, either as a result of its innate chemical structure and/or the lower dissociation enthalpy/entropy and higher solubility of DBS‐CONHNH_2_‐containing gels reflecting more dynamic materials at the molecular level, that may be less able to provide focal adhesion to the hMSCs. These results provide valuable insights that contribute to important, ongoing efforts to develop simple design principles for supramolecular gels as hMSC scaffolds.^[^ ^56^ ^]^

In future work, we intend to fully exploit the potential of DBS‐CH_2_OH as a new cytocompatible LMWG with the goal of developing systems with clinical relevance and fabricating shaped and patterned multi‐component gels that direct stem cell growth in sophisticated ways. We suggest that the spread hMSCs obtained on these simple DBS‐CH_2_OH gels, remarkably different from the rounded cells observed on gels containing DBS‐CONHNH_2_, indicate excellent cell adhesion and have particular promise in terms of controlling osteogenesis.

## Conflict of Interests

The authors declare no conflict of interest.

## Supporting information



Supporting information

## Data Availability

The data that support the findings of this study are available in the supplementary material of this article.
