# Supplementary material for: Molecular‐Scale Tuning of Low‐Molecular‐Weight Gelators Controls Supramolecular Assembly and Directs Human Mesenchymal Stem Cell Growth
Source: Angew Chem Int Ed Engl. 2026 Jan 24;65(10):e23454. doi: 10.1002/anie.202523454 (PMC12955537; doi:10.1002/anie.202523454)
Supplement: Supplementary file 1 — Supporting information [file ANIE-65-e23454-s001.docx]

**Molecular-Scale Tuning of Low-Molecular-Weight Gelators Controls Supramolecular Assembly and Directs Human Mesenchymal Stem Cell Growth**

Chayanan Tangsombun,^a^ Amy Simpson,^b^ Rebecca L. Charlton,^a^ Lucy E. Sabin,^a^ Paul G. Genever^b^ and David K. Smith*^,a^

a: Department of Chemistry, University of York, Heslington, York, YO10 5DD, UK

b: Department of Biology, University of York, Heslington, York, YO10 5DD, UK

**SUPPLEMENTARY INFORMATION**

1. General Experimental Methods
2. Synthesis and Characterization of DBS-CH_2_OH
3. Gel Fabrication
4. ^1^H-NMR
5. Circular Dichroism
6. Scanning Electron Microscopy
7. Transmission Electron Microscopy
8. Thermal Stability Studies
9. Rheology Studies
10. Infrared Spectroscopy
11. Biological Studies
12. References

**S1 General Experimental Methods**

Chemicals were purchased from standard chemical suppliers for synthesis and analysis. DBS-COOMe and DBS-CONHNH_2_ were prepared as described in our previous reports.^1,2^ All characterisation data were in agreement with previous reports. ^1^H NMR were recorded on a Jeol 400 spectrometer (^1^H 400 MHz). Samples were prepared as solutions in deuterated NMR solvents (DMSO-d_6_) and chemical shifts (δ) are quoted in parts per million (ppm). ^1^H NMR studies with variable temperature were performed on Unshielded 500 MHz spectrometer (an AVIII Console with a BBI 500S2 H&F-BB-D-05 probe, part number Z120379_0002, running topspin 3.7.0). Mass spectrometry was performed on the ESI Bruker Daltonics Micro-TOF Mass spectrometer by the University of York Mass Spectrometry Service. Circular dichroism (CD) spectra were recorded on a Jasco J-1500 spectrophotometer. IR spectra were measured on a PerkinElmer Spectrum Two FT-IR spectrometer. TEM images were recorded on a Zeiss Gemini 460 Field Emission Gun Scanning Electron Microscope (FEGSEM) with using a STEM (Scanning Transmission Electron Microscopy) detector. SEM images were obtained from the Zeiss Gemini 460 FEGSEM with an In-Lens SE1 detector. Fibre sizes from electron microscopy were determined using *ImageJ* software. *T_gel_* values were obtained using a high-precision thermoregulated oil bath. Rheological measurements were recorded using a Malvern Instruments Kinexus Pro+ rheometer fitted with a 20 mm parallel plate geometry. Fluorescence measurements for the cell viability assay were performed using a BMG Labtech Clariostar Plate Reader. The fluorescence images of control gels stained with calcein AM and propidium iodide were collected using a Zeiss AXIO Observer 7 inverted Fluorescence motorized XYZ Definite Focus Microscope. Confocal images were obtained using Zeiss LSM 880 with Airyscan.

**S2 Synthesis and Characterization of DBS-CH_2_OH**

DBS-COOMe (0.50 g, 1.054 mmol) was added into a two-necked round-bottom flask fitted with a dropping funnel and condenser. Under N_2_ atmosphere, dry THF (25 mL) was added, and the suspension stirred for 15 min. The mixture was cooled to 0 °C, and LiAlH_4_ (18 mL, 1 M in THF) was added dropwise through a dropping funnel to the reaction. The mixture was refluxed at 70 °C for 8 h under N_2_ atmosphere then left at room temperature for 12 h. After that period, the reaction was cooled to 0 °C and a second portion of LiAlH_4_ (1 mL, 1 M in THF) was injected. The reaction was then refluxed for 6 h until there was no DBS-COOMe on TLC using CH_2_Cl_2_:MeOH (4:1) as mobile phase. The reaction was then cooled to 0 °C and quenched by dropwise addition of EtOAc (*ca.* 6 mL), followed by careful addition of water (150 mL). This mixture was left to stir for 30 min. The white paste was collected and dried in an oven overnight. To purify the crude product, methanol (50 mL) was added to the flask with stirring being continued for 15 min at 50 °C. The filtrate was collected, and the purification process was repeated four times. The combined filtrate was then concentrated on a rotary evaporator and deionized water (50 mL) was added. This mixture was acidified to pH 7 with NaHSO_4_ (1.5 M) The resulting product was filtered and washed with deionized water to obtain a white solid. The product was dried by air and then in a vacuum oven to obtain a constant mass of DBS-CH_2_OH (0.30 g, 68 %).


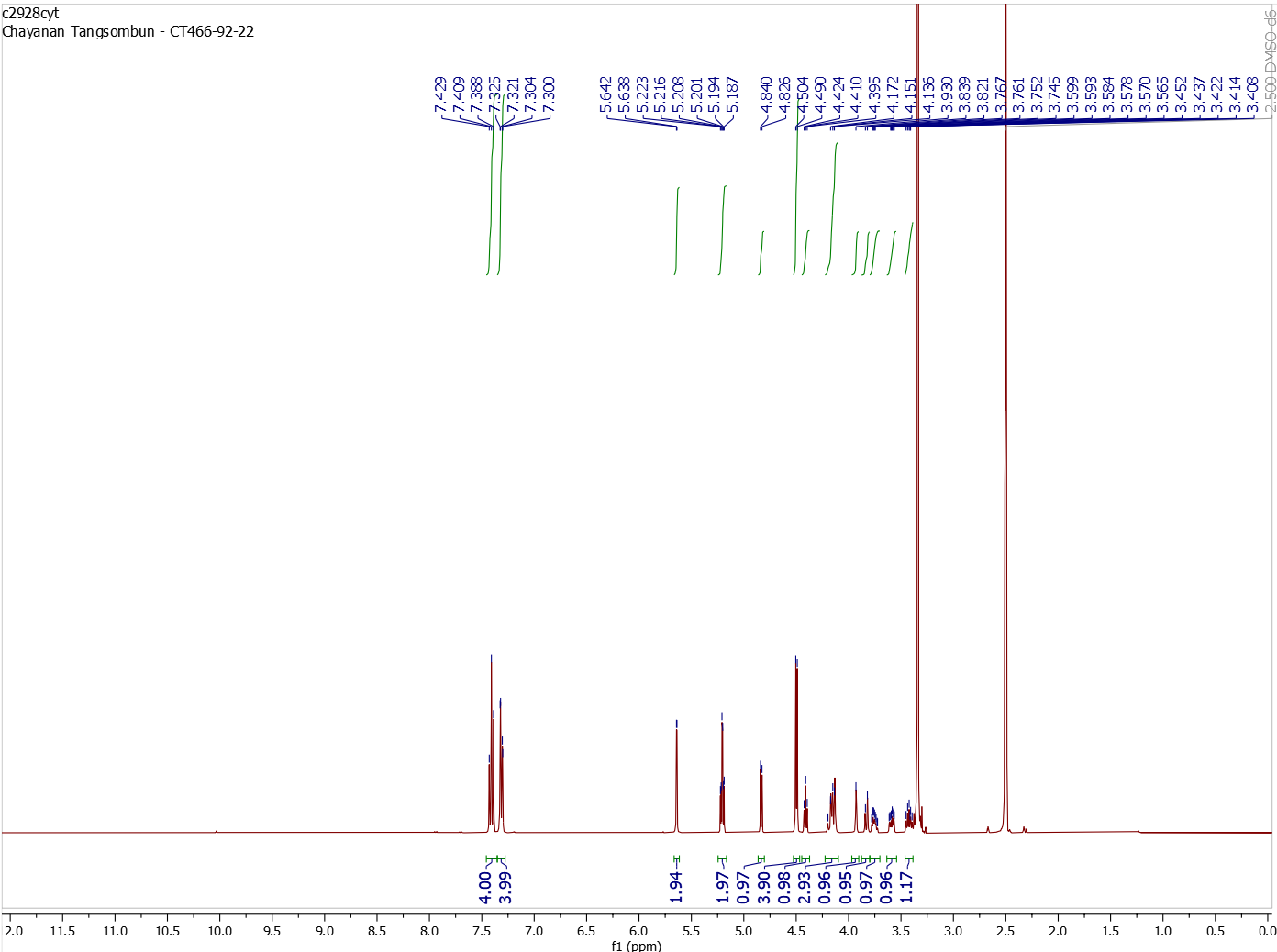
^1^H NMR (400 MHz, DMSO-d6): δ 7.43-7.37 (m, 4H, Ar*H*), 7.32-7.29 (m, 4H, Ar*H*), 5.64 (s, 2H, Ar-C*H*), 5.20 (2t, 2H, *J* = 5.6 Hz, Ar-CH_2_-O*H*), 4.83 (d, 1H, *J* = 6.0 Hz, CHO*H*), 4.50 (d, 4H, *J* = 5.6, Ar-C*H_2_*-OH), 4.41 (t, 1H, *J* = 5.6, CH_2_O*H*), 4.20-4.12 (m, 3H (overlap), sugar), 3.93 (s, 1H, sugar), 3.85-3.81 (m, 1H, sugar), 3.78-3.72 (m, 1H, sugar), 3.62-3.56 (m, 1H, sugar), 3.46-3.38 (m, 1H, sugar). ^13^C NMR (400 MHz, DMSO-d6): δ 142.99 (aromatic *p*-C), 142.90 (aromatic *p*-C), 137.19 (aromatic C), 136.93 (aromatic C), 125.94 (aromatic *o*-C), 125.92 (aromatic *o*-C), 125.87 (aromatic *m*-C, overlap), 99.38 (Ph-CH), 99.32 (Ph-CH), 77.67 (CH), 70.11 (CH), 69.33 (CH_2_), 68.45 (CH), 67.71 (CH), 62.64 (benzyl CH_2_ overlapped, *CH_2_*-OH sugar). ESI-MS (m/z) calc. for C_22_H_26_NaO_8_ 441.1520; found 441.1515 ([M+Na])^+^. IR ν_max_ (cm^-1^) (solid): 3207m, 2981w, 2957w, 2941w, 2864w, 1460w, 1450w, 1419m, 1400m, 1371m, 1342m, 1329m, 1312w, 1265w, 1246w, 1223m, 1167m, 1133w, 1120w, 1090s, 1068m, 1052s, 1045s, 1016vs, 1006vs, 981s, 954m, 947m, 920w, 902w, 884m, 834s, 792m, 777m, 762s, 742m, 713m, 661s, 637m, 620m, 599m, 579s, 551s, 530m, 525m, 501m, 465m. Melting Point: 234-236 °C.

Figure S1. **^1^**H NMR spectrum of DBS-CH_2_OH in DMSO-d6 (400 MHz).


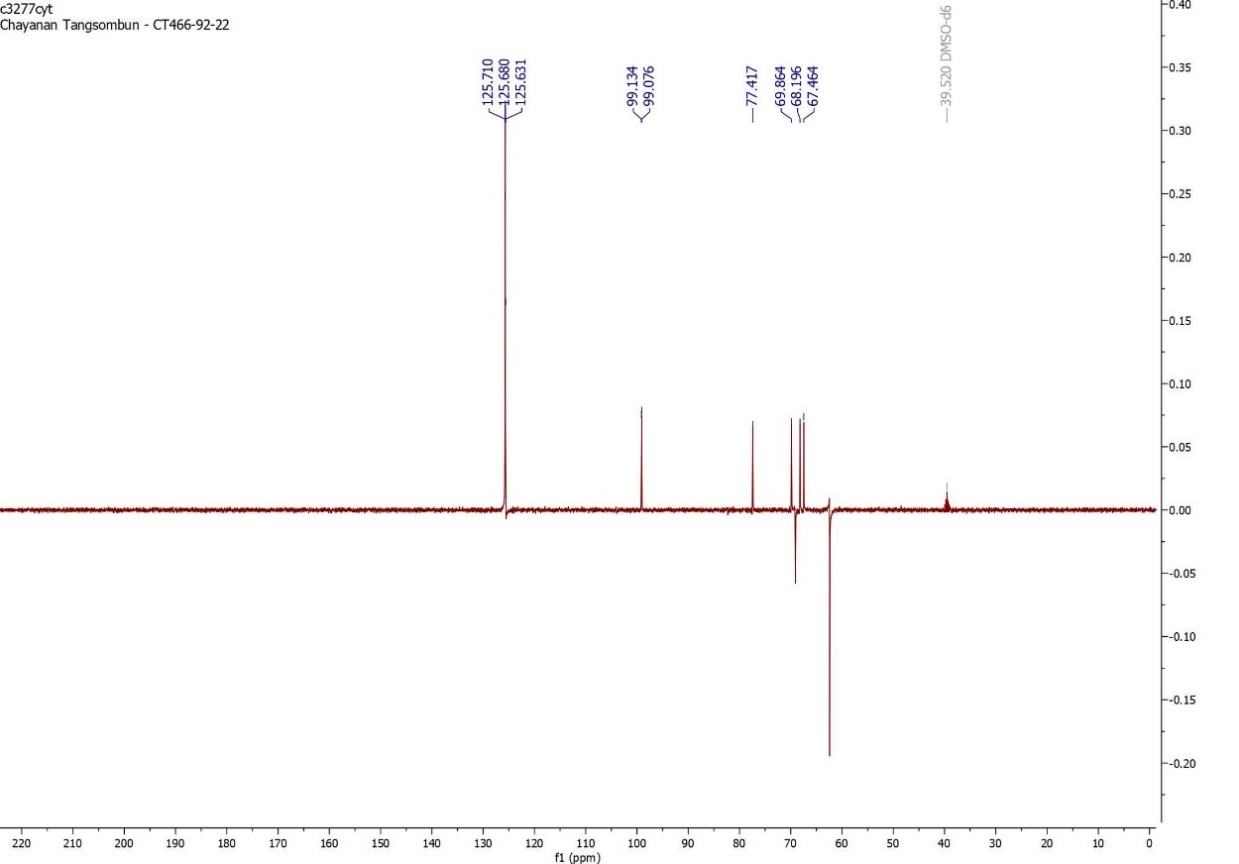


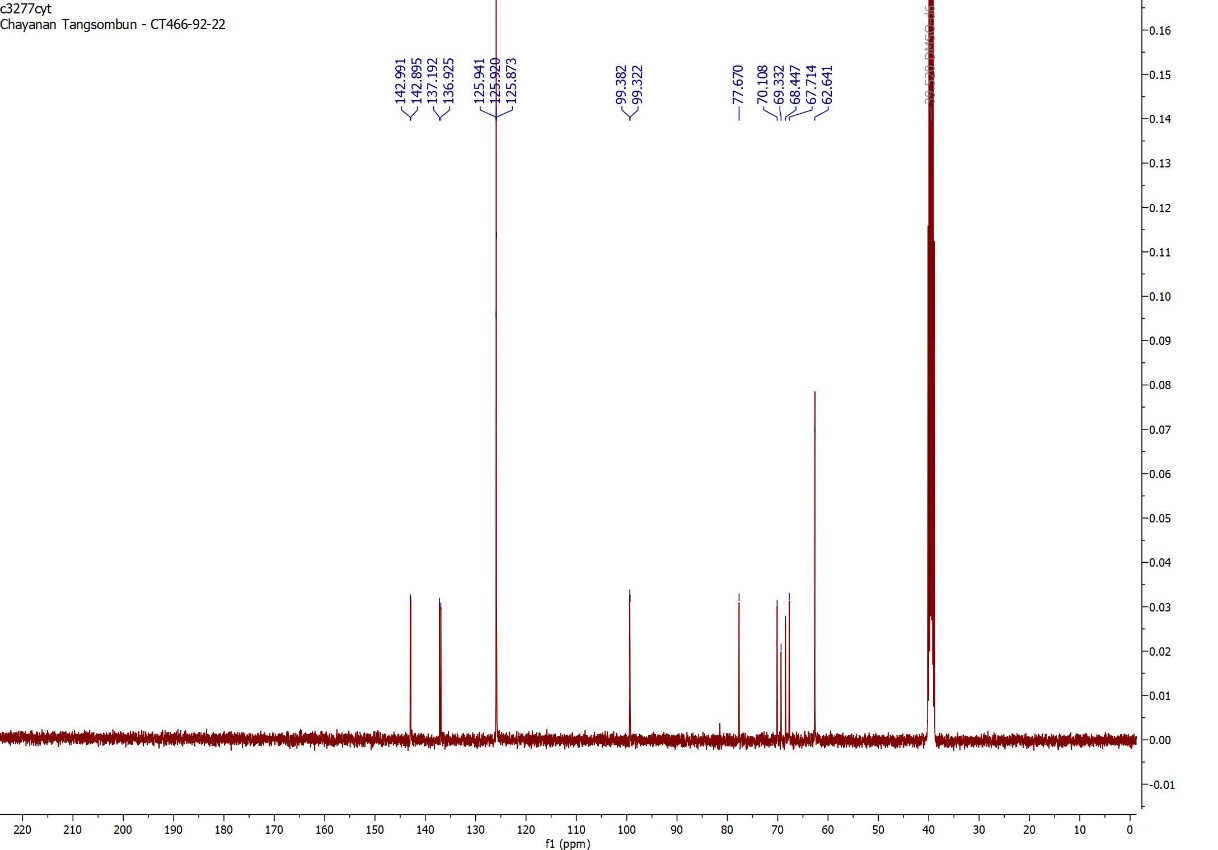
Figure S2. DEPT135 spectrum of DBS-CH_2_OH in DMSO-d6 (400 MHz).

Figure S3. ^13^C NMR spectrum of DBS-CH_2_OH in DMSO-d6 (400 MHz).


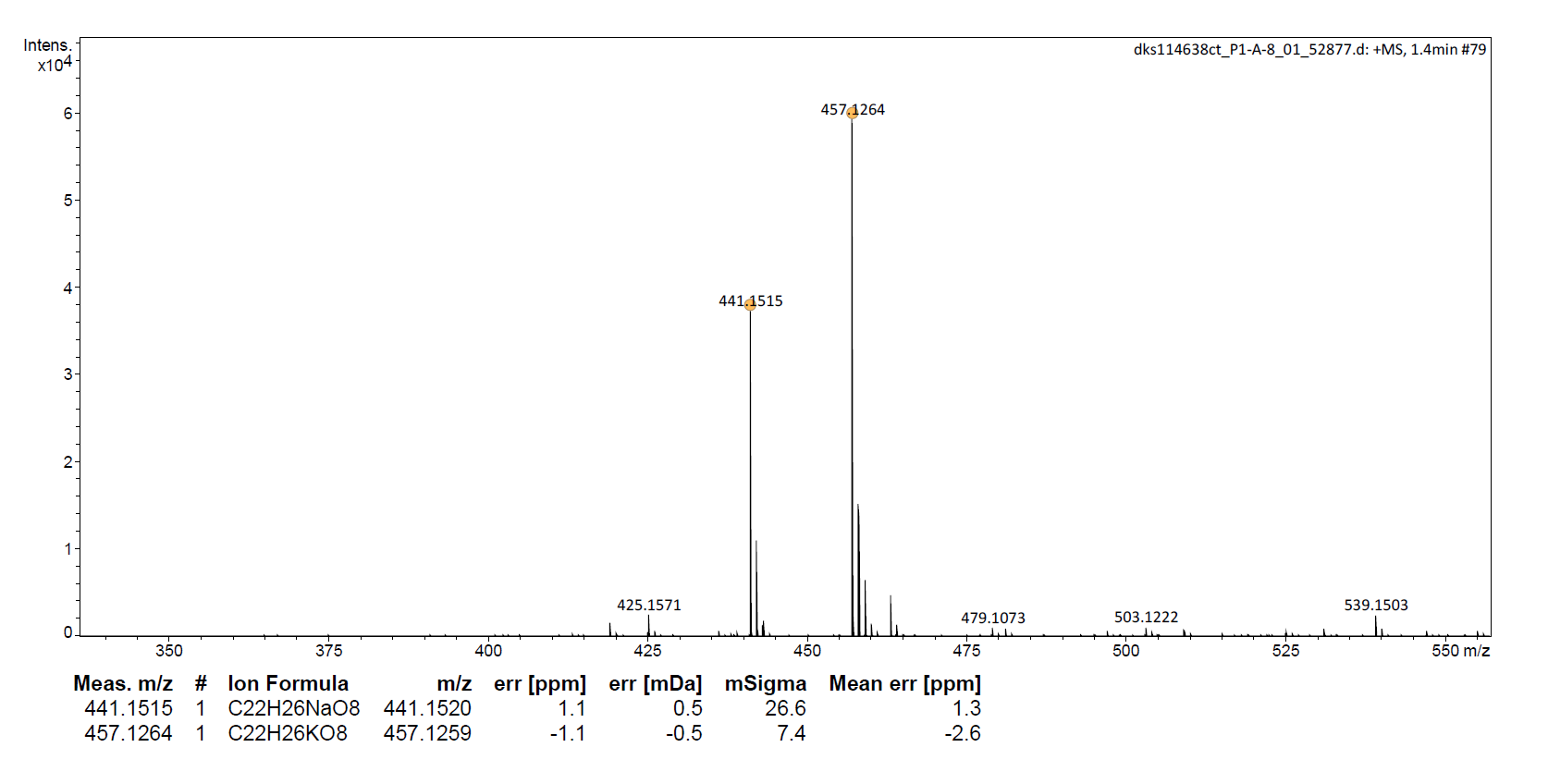


Figure S4. Mass spectrum of DBS-CH_2_OH.

**S3** **Gel Fabrication**

**S3.1 Preparation of DBS-CH_2_OH gels.** A known amount of DBS-CH_2_OH was weighed in a screw cap vial. Water (1 mL) was added and sonicated (*ca*. 30 min) to obtain a fine suspension. This was then heated with a heat gun until complete dissolution and left undisturbed overnight to allow gel formation.

**S3.2 Preparation of DBS-CONHNH_2_ gels.** A known amount of DBS-CONHNH_2_ was suspended in water (1 mL) and sonicated. This mixture was heated with a heat gun until complete dissolution and left undisturbed overnight to allow gel formation.

**S3.3 Preparation of DBS- CH_2_OH/DBS-CONHNH_2_ gels.** A known amount of DBS-CONHNH_2_ was suspended in water (1 mL) and sonicated. The mixture was transferred to a vial containing a known amount of DBS-CH_2_OH and sonicated. The sample was heated with a heat gun and left overnight to allow gel formation.

**S4** **^1^H NMR Experiments**

**S4.1 Room Temperature Studies.** ^1^H NMR samples were prepared as described in Section 3, using D_2_O. DBS-CH_2_OH (0.2 or 0.3% wt/vol), DBS-CONHNH_2_ (0.3% wt/vol), DBS-CH_2_OH/DBS-CONHNH_2_ (0.15 or 0.3% wt/vol for each) or DBS-CH_2_OH/DBS-CONHNH_2_ (0.2 wt/vol for DBS-CH_2_OH and 0.3% wt/vol for DBS-CONHNH_2_) in D_2_O (0.7 mL) was heated to complete dissolution. DMSO (1.4 μL) as internal standard was then added into the samples before they were transferred to an NMR tube. The samples were left
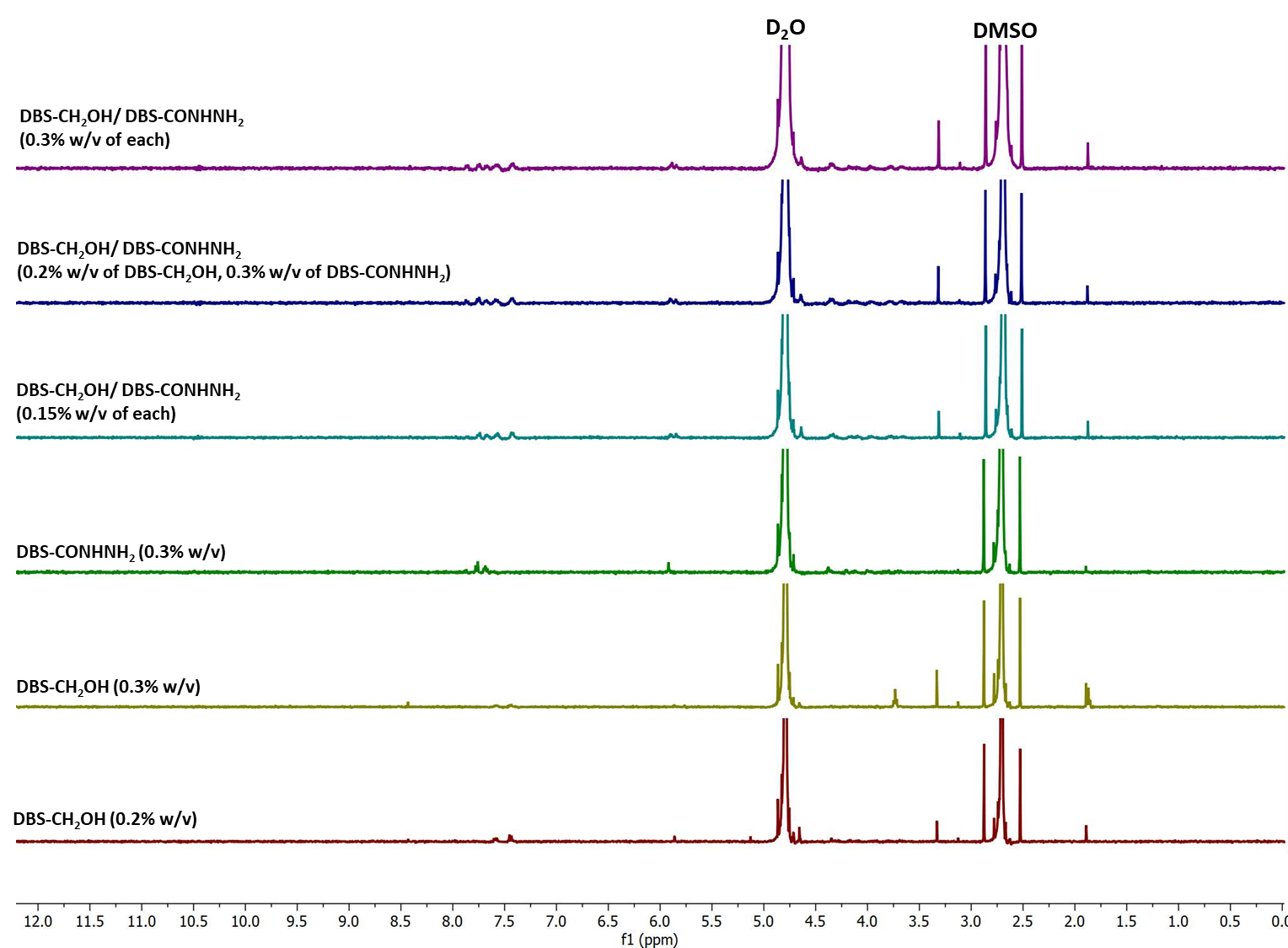
overnight to allow gel formation, and ^1^H NMR spectra were recorded (Figure S6.5).

Figure S5. **^1^**H NMR studies at room temperature of DBS-CH_2_OH, DBS-CONHNH_2_ and DBS-CH_2_OH/DBS-CONHNH_2_ multicomponent gels using different concentrations prepared in D_2_O with DMSO as internal standard.

**S4.2 Variable Temperature Experiments.** DBS-CH_2_OH (0.3% wt/vol), DBS-CONHNH_2_ (0.3% wt/vol) and DBS-CH_2_OH/DBS-CONHNH_2_ (0.15% wt/vol of each) were prepared in D_2_O (0.7 mL) with addition of DMSO (1.4 μL) as internal standard, as described above. Spectra were recorded using a 500 MHz spectrometer (an AVIII Console with a BBI 500S2 H&F-BB-D-05 probe, part number Z120379_0002, running topspin 3.7.0.) In all cases, the first spectrum for each sample was recorded at 25 °C. The samples were heated to 60 °C and held for 15 min before acquisition of the second spectrum, and then the equilibration time was reduced to 10 minutes for the subsequent 5 °C jumps, until 85°C.

Figure S6. ^1^H NMR spectra of DBS-CONHNH_2_ (0.30% wt/vol) recorded at different temperatures.

Figure S7. ^1^H NMR spectra of DBS-CH_2_OH (0.30% wt/vol) recorded at different temperatures.

Figure S8. ^1^H NMR spectra of multi-component DBS-CH_2_OH/DBS-CONHNH_2_ (0.15% wt/vol of each) recorded at different temperatures.

**S5 Circular Dichroism**


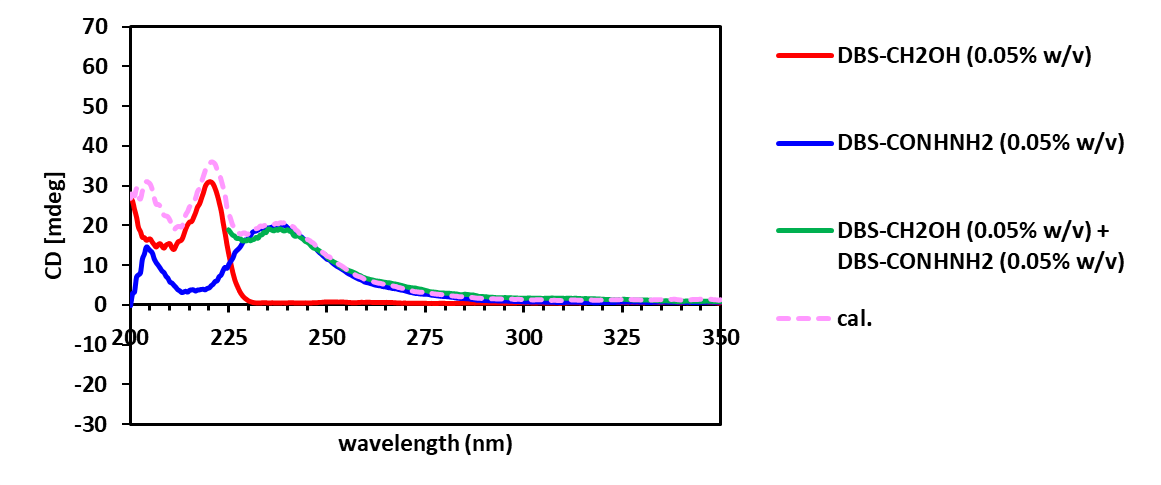
All CD spectra were measured at 20 °C on a Jasco J-1500 spectropolarimeter between 200 and 400 nm using the following settings: Data Pitch = 0.5 nm, Bandwidth = 1.00 nm, Scanning Mode = continuous, Scan Speed = 100 nm/min and accumulation = 5. A CD cuvette (quartz, pathlength 1 mm) was used. For variable temperature experiments, measurements were carried out at 5 °C intervals from 20-90 °C.

Figure S9. CD spectra at 90 °C of DBS-CH_2_OH, DBS-CONHNH_2_, multicomponent DBS-CH_2_OH/DBS-CONHNH_2_ and theoretical line calculated from CD spectra of each component at 90 °C.

**DBS-CONHNH_2_ or DBS-CH_2_OH:** Water (4 mL) was added to a vial containing a known mass of gelator (1.6 mg for 0.04% wt/vol, 2.0 mg for 0.05% wt/vol or 2.8 mg for 0.07% wt/vol). The mixture was sonicated and then heated with a heat gun until complete dissolution. The solution (0.4 mL) was transferred to a CD cuvette and left for 4 h before spectra were recorded.

**DBS-CH_2_OH/ DBS-CONHNH_2_:** A suspension of DBS-CONHNH_2_ (4 mL, 0.04, 0.05 or 0.07% wt/vol) was transferred to a vial containing DBS-CH_2_OH (1.6, 2.0 or 2.8 mg), sonicated and heated with a heat gun until complete dissolution.

**S6 Scanning Electron Microscopy**

All samples were imaged using a Zeiss Gemini 460 Field Emission Gun Scanning Electron Microscope (FEGSEM). Topography rich secondary electron micrographs were obtained in high vacuum mode, at 3kV accelerating voltage and between 16 pA and 24 pA probe current at working distances of less than 4.5 mm using an In-Lens SE1 detector. Gel samples were prepared for secondary electron imaging by depositing a thin smear of gel on a small (ca. 5 mm x 5 mm) copper shim, followed by plunge freezing in slushy nitrogen and freeze drying using a Polaron E6000 series vacuum coater equipped with a Peltier freeze drying stage. The freeze-dried samples were mounted on 10 mm aluminium pin stubs by means of self-adhesive carbon-rich discs, excess material was knocked off the upper surfaces using stainless steel forceps and the mounted samples were sputter coated with 8 nm of platinum using a Safematic CCU-010 high resolution sputter coater.


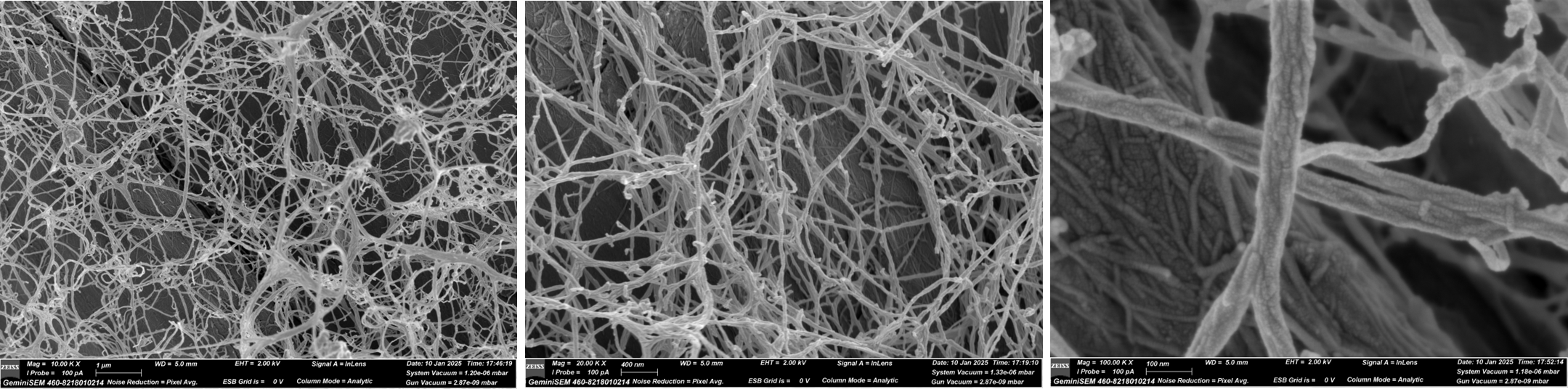
Figure S10. SEM images of DBS-CH_2_OH hydrogel (0.3% wt/vol), Scale bar: 1 μm (left), 400 nm (middle) and 100 nm (right).


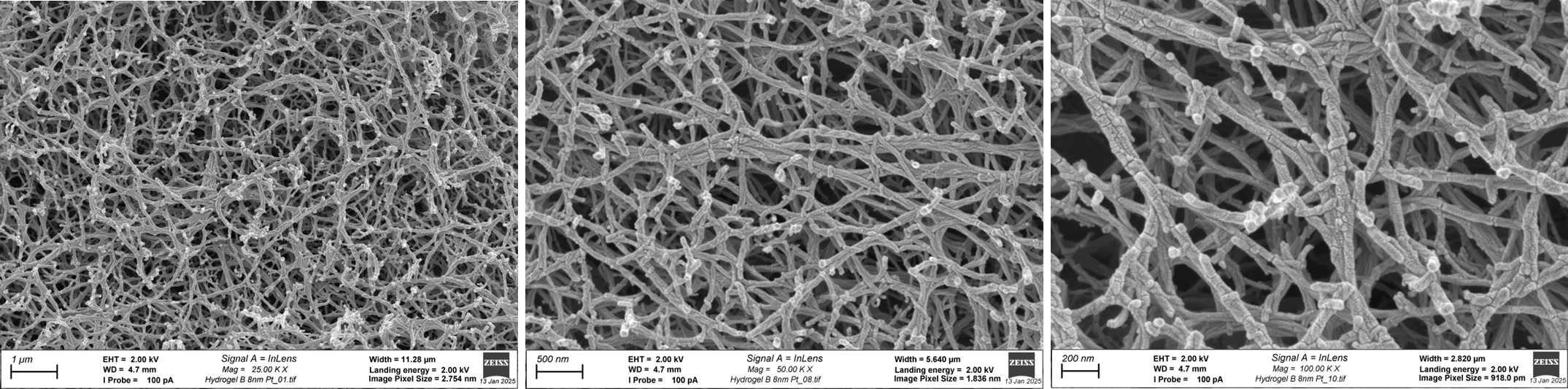
Figure S11*.* SEM images of DBS-CONHNH_2_ hydrogel (0.3% wt/vol), Scale bar: 1 μm (left), 500 nm (middle) and 200 nm (right).


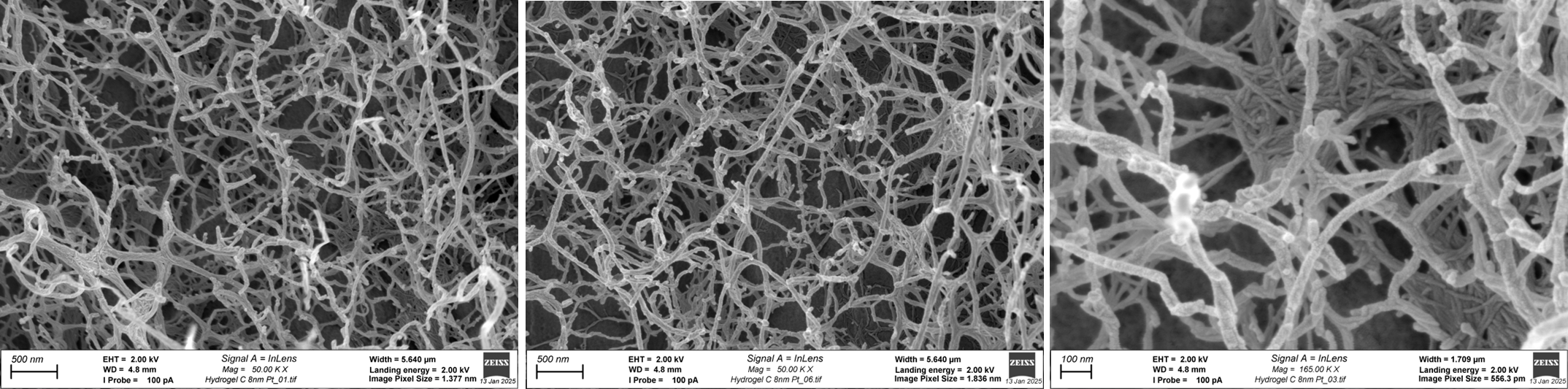
Figure S12*.* SEM images of DBS-CH_2_OH/DBS-CONHNH_2_ hydrogel (0.3% wt/vol), Scale bar: 1 μm (left), 500 nm (middle) and 200 nm (right)*.*

**S****7 Transmission Electron Microscopy**

Transmission electron micrographs were obtained by mobilising the gels by shear using a whirlimixer for approximately 10 seconds and pipetting 3 µl of hydrogel onto formvar/carbon coated 200 mesh copper TEM grids (Gilder, square mesh), allowing them to adsorb for 60 seconds and wicking off the excess material into a torn edge of Whatman No. 1 filter paper. The wicked off grids were immediately negatively stained using 2% wt/wt aqueous uranyl acetate; pipetting 10 µl of stain onto the sample/grid surface and immediately wicking off with torn filter paper to effect flash drying of the sample grids in readiness for imaging in the FEGSEM. Micrographs were obtained using a STEM (Scanning Transmission Electron Microscopy) detector in the Gemini 460 FEGSEM at the following conditions: high vacuum mode at 30 kV accelerating voltage, 16 pA probe current and 8.3 mm to 8.5 mm working distance.

**
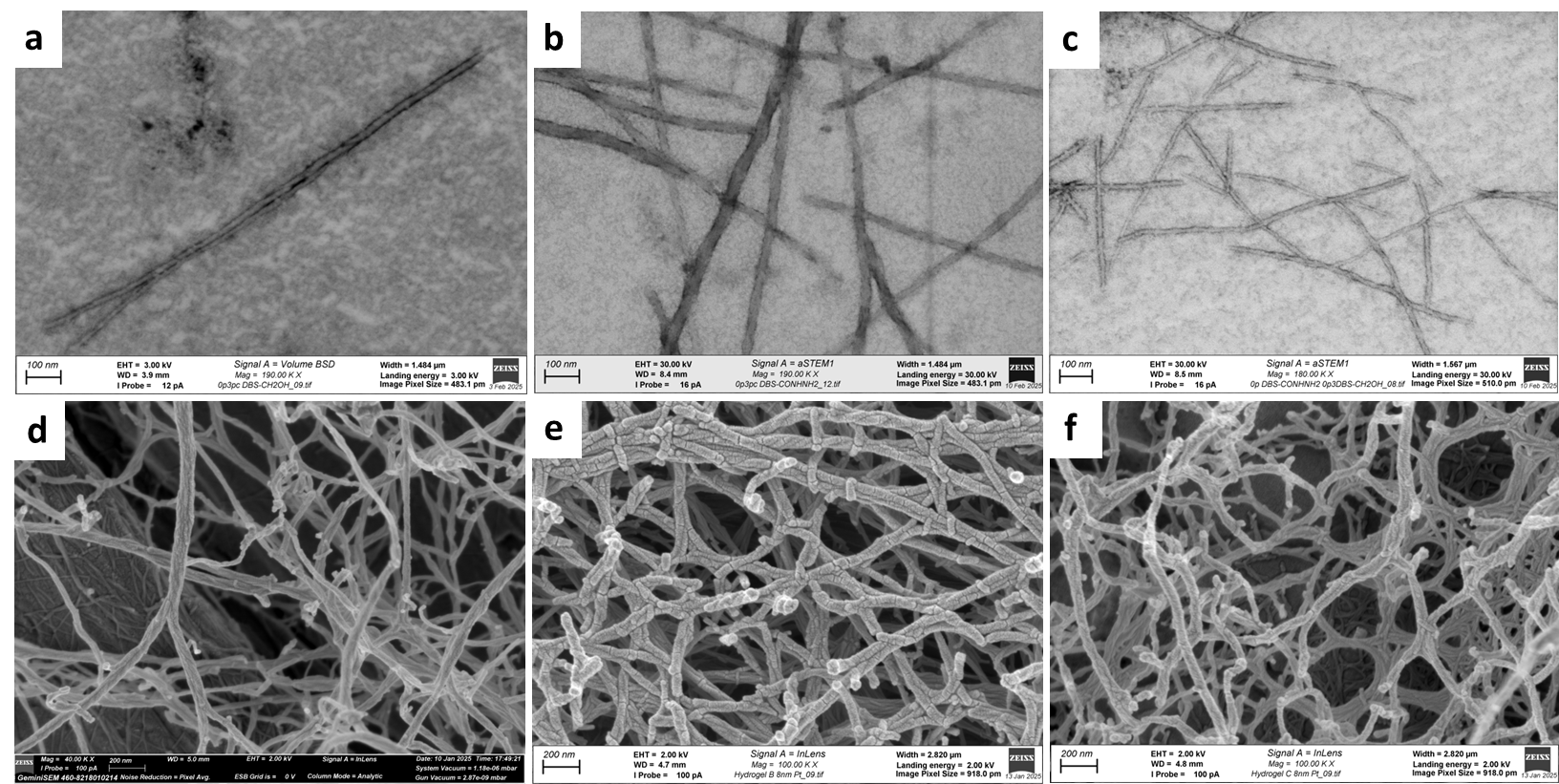
**Figure S13. TEM (top) and SEM (below) images of DBS-CH_2_OH (0.3% wt/vol), DBS-CONHNH_2_ (0.3% wt/vol) and DBS-CH_2_OH/DBS-CONHNH_2_ (0.3% wt/vol of each) multicomponent hydrogels.

**
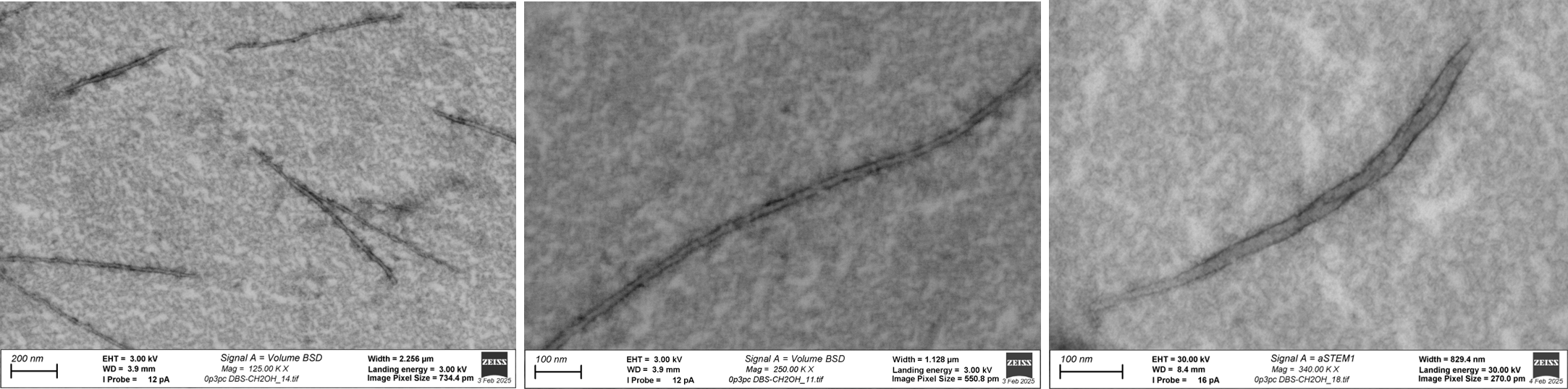
**Figure S14*.* Additional TEM images of DBS-CH_2_OH hydrogel (0.3% wt/vol), Scale bar: 200 nm (left) and 100 nm (middle, right)*.*

**
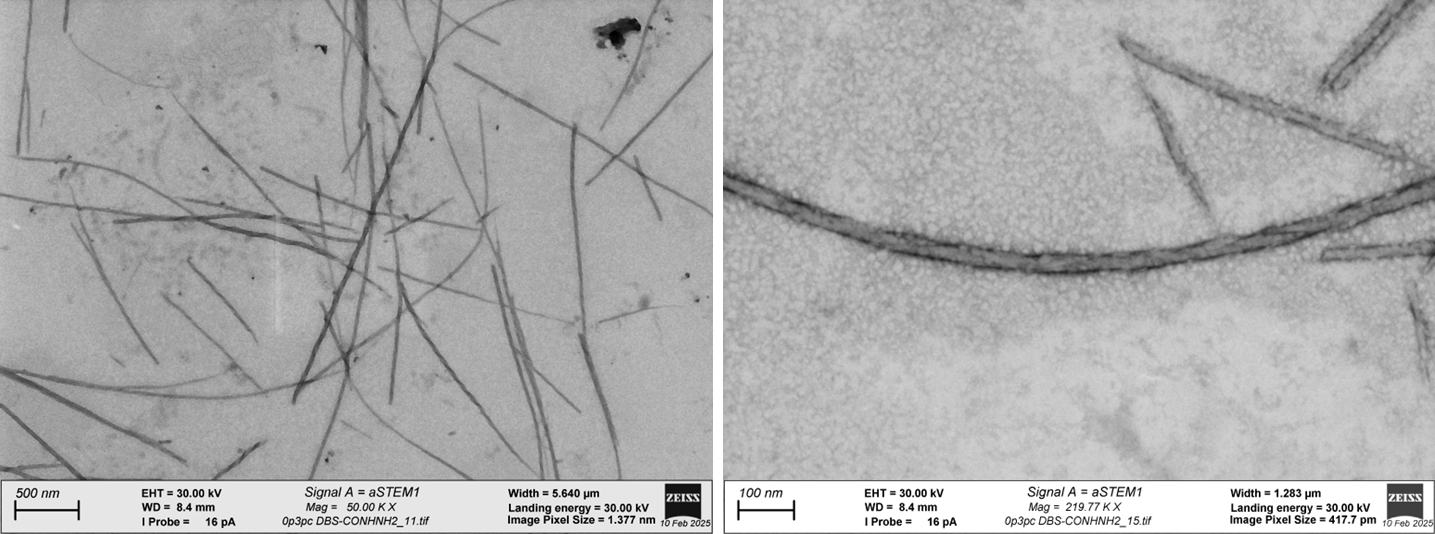
**

Figure S15. Additional TEM images of DBS-CONHNH_2_ hydrogel (0.3% wt/vol), Scale bar: 500 nm (left) and 100 nm (right).

**
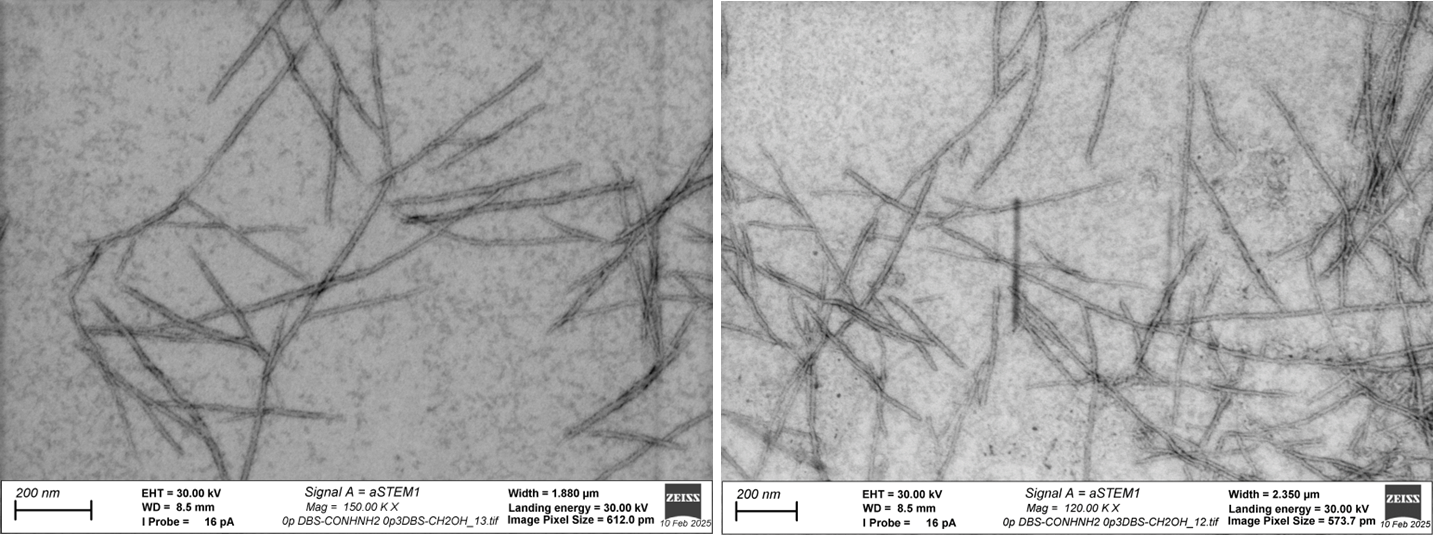
**Figure S16. Additional TEM images of DBS-CH_2_OH/DBS-CONHNH_2_ hydrogel (0.3% wt/vol of each LMGW), Scale bar: 500 nm (left) and 100 nm (right).

**S8 Thermal Stability Studies**

All the gels were prepared as described in Section S3 in 7 ml vials (diameter = 2 cm, height = 6 cm), then placed in a high precision thermoregulated oil bath with an initial temperature of 20 °C. The temperature was increased by 1 °C/min until 100 °C. The integrity of the gels was checked by tube inversion every minute. The temperature (*T*_gel_) was recorded when the gel began to run down the side of the vial. These experiments were performed in triplicate and the average *T*_gel_ is recorded. Errors are estimated at ± 2°C.

**S9 Rheology Studies**

Gels were prepared as described in Section S3 in bottomless vials. Gels (1 mL total) were placed on the rheometer, and measurements were carried out at 25 °C using a 20 mm parallel plate with a gap of 2 mm. The amplitude sweep was performed in the range of 0.01-100% strain at a frequency of 1 Hz. The frequency sweep was performed between 0.1-100 Hz using a shear strain of 0.10%. For reproducibility, the experiments were performed in triplicate, and average data were used to plot the graphs.


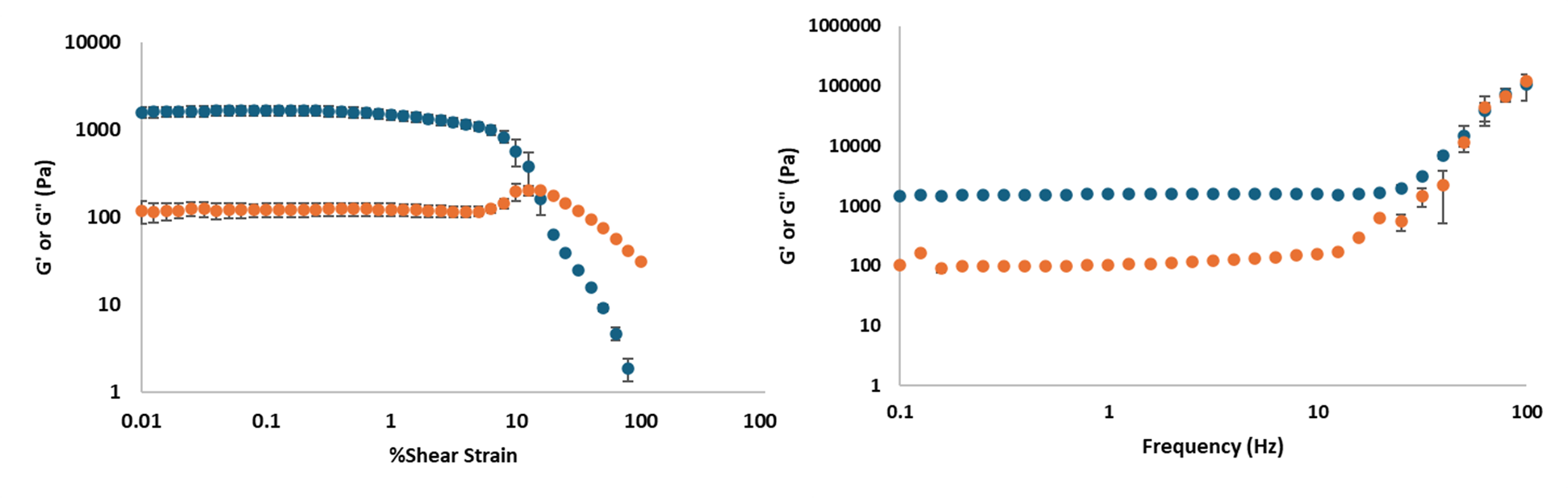


Figure S17. Elastic (G’, blue circles) and viscous (G”, orange circles) moduli of DBS-CH_2_OH (0.2% wt/vol) hydrogel with increasing shear strain (left) and frequency (right).


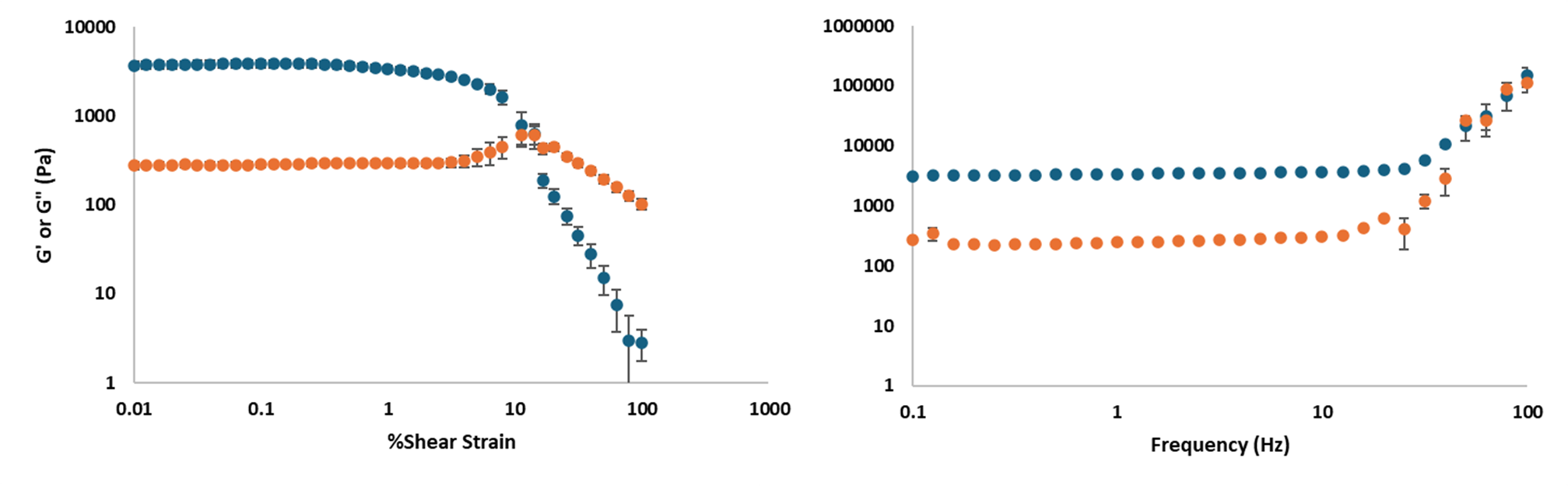


Figure S18. Elastic (G’, blue circles) and viscous (G”, orange circles) moduli of DBS-CH_2_OH (0.3% wt/vol) hydrogel with increasing shear strain (left) and frequency (right).


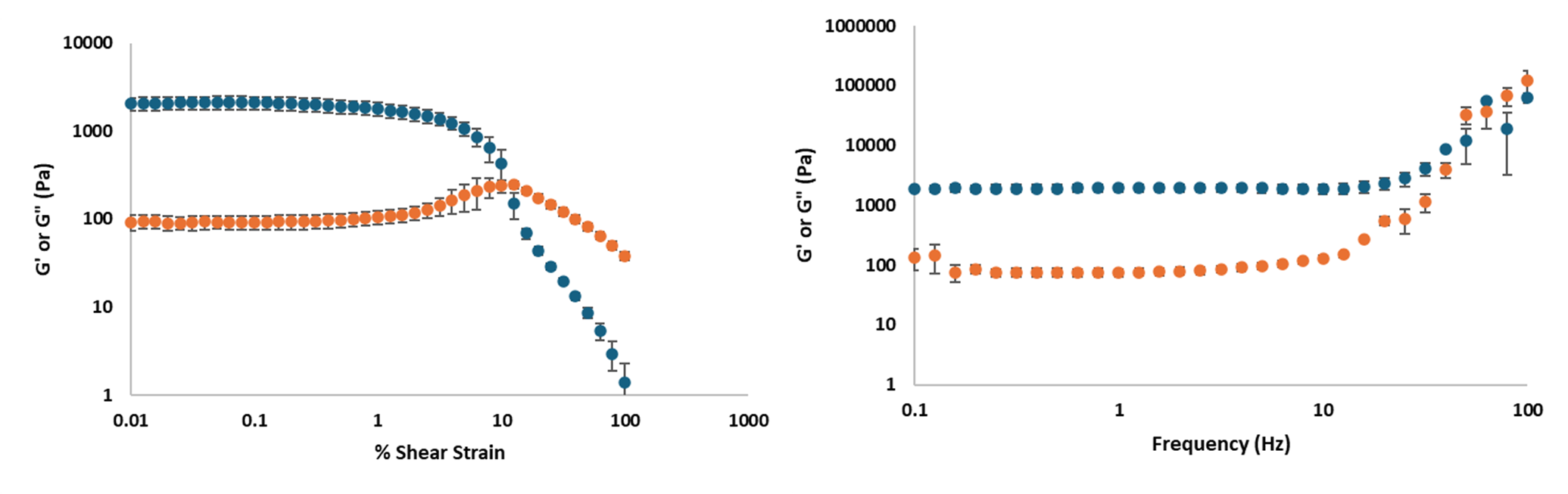


Figure S20. Elastic (G’, blue circles) and viscous (G”, orange circles) moduli of DBS-CH_2_OH/DBS-CONHNH_2_ (0.15% wt/vol of each) hydrogel with increasing shear strain (left) and frequency (right).


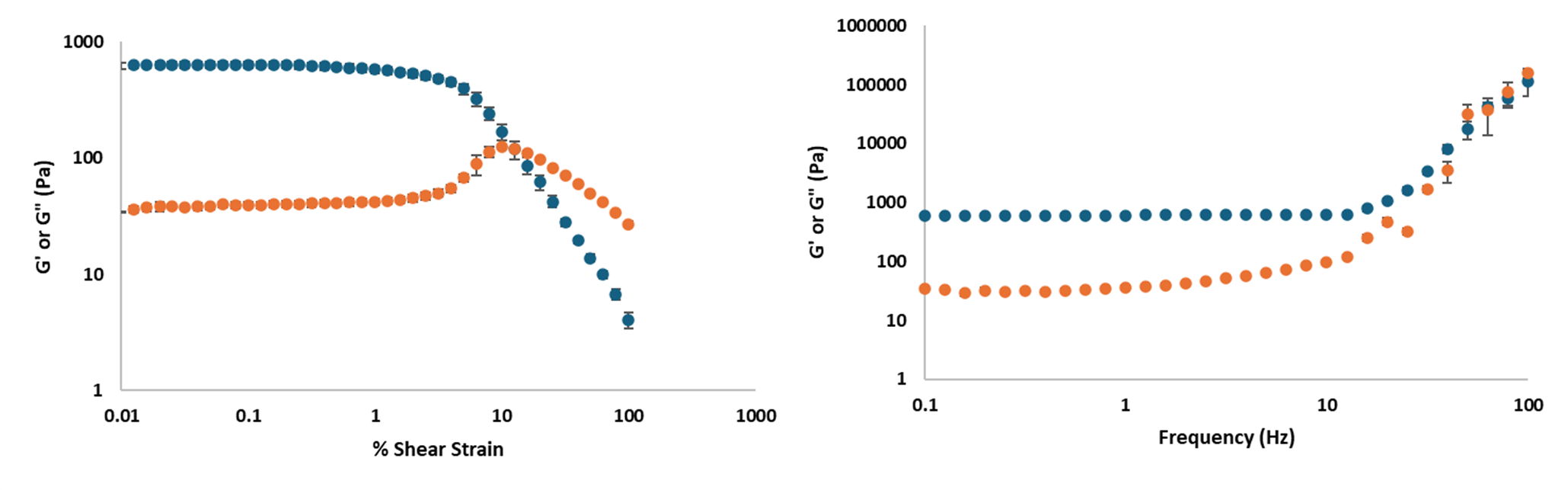


Figure S19. Elastic (G’, blue circles) and viscous (G”, orange circles) moduli of DBS-CONHNH_2_ (0.3% wt/vol) hydrogel with increasing shear strain (left) and frequency (right).


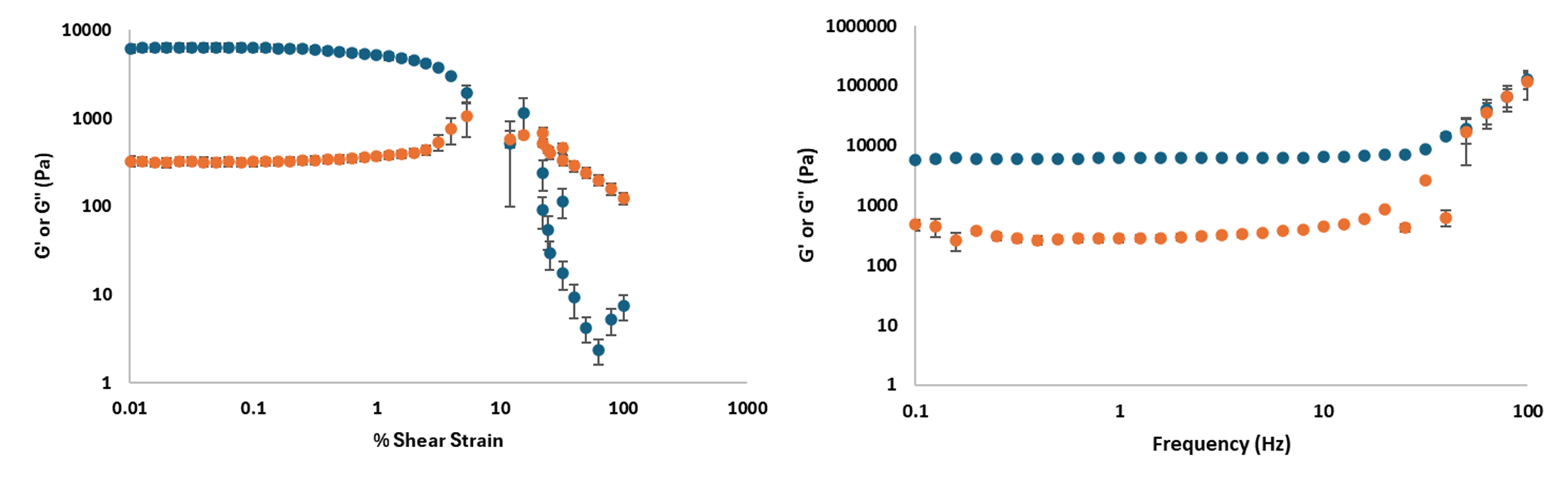


Figure S21. Elastic (G’, blue circles) and viscous (G”, orange circles) moduli of DBS-CH_2_OH/DBS-CONHNH_2_ (0.2% wt/vol of DBS-CH_2_OH and 0.3% wt/vol of DBS-CONHNH_2_) hydrogel with increasing shear strain (left) and frequency (right).


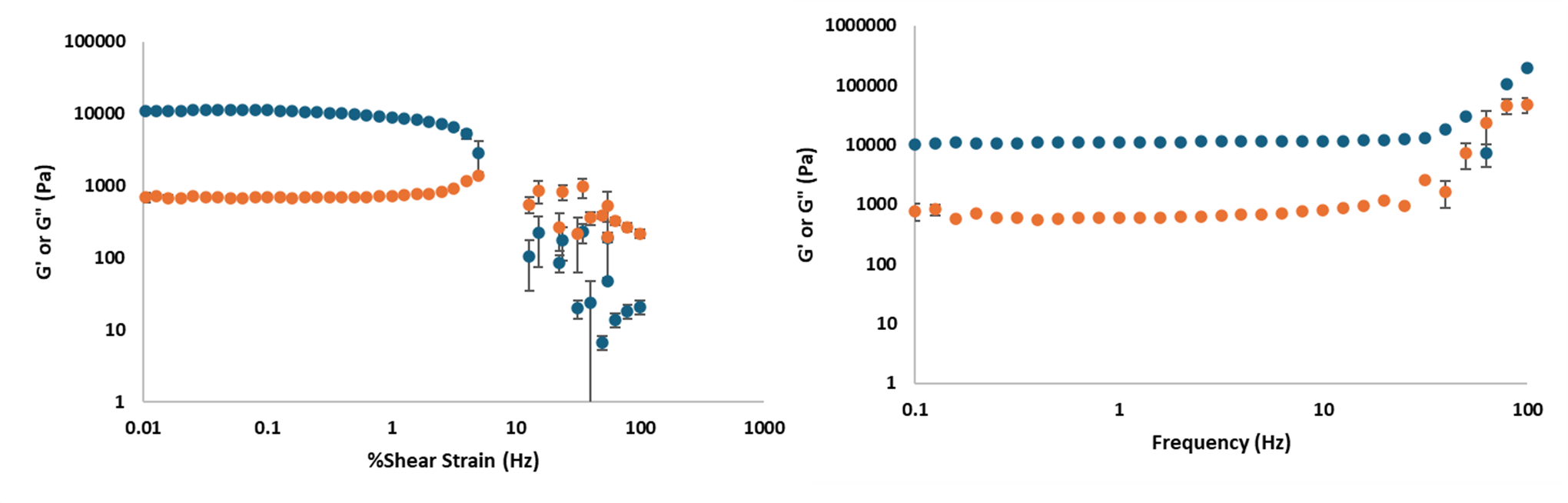


Figure S22. Elastic (G’, blue circles) and viscous (G”, orange circles) moduli of DBS-CH_2_OH/DBS-CONHNH_2_ (0.3% wt/vol of each) hydrogel with increasing shear strain (left) and frequency (right).

**Stress relaxation experiments.** Gels were placed on the rheometer, and measurements were carried out at 37 °C using a 20 mm parallel plate with a gap of 2 mm. Gels were allowed to equilibrate to 37 °C  for 5 minutes on the plate. In each case, a constant strain within the LVR was rapidly applied to the gel (1.0% for DBS-CO_2_H and DBS-CONH_2_, 0.5% for the two-component gel). The stress relaxation experiment was run for 5 minutes. In each case, we found that the stress rapidly (<0.1 s) relaxed to a minimum and then the gel built up to the expected G’ value within 1 s. As such, we determine that bulk stress relaxation is rapid in all of these gels.


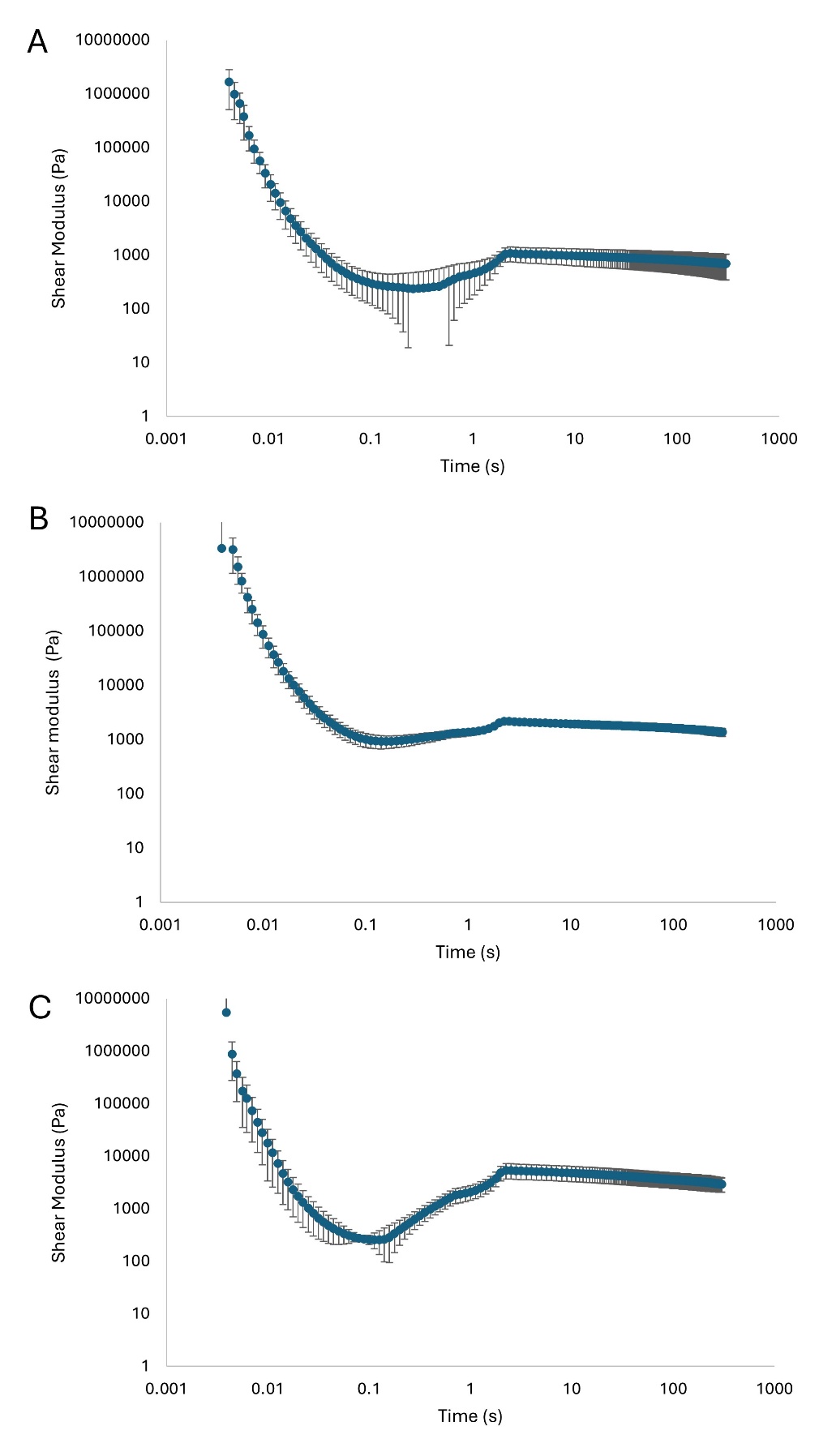


Figure S23. Stress relaxation plots for gels based on (A) DBS-CONHNH_2_ (0.3% wt/vol), B DBS-CH_2_OH (0.3% wt/vol), and (C) DBS-CONHNH_2_ and DBS-CH_2_OH (both 0.3% wt/vol).

**S10 Contact Angle Measurements**

Contact angle measurements were obtained on an Ossila contact angle goniometer, using a liquid drop of water (10 μL) using the sessile drop method, with a high-res camera capturing images for analysis of wetting properties. In all cases, the water droplet immediately spread over and into the surface of the gel, preventing an actual contact angle measurement, and indicating that, as expected, these are very hydrophilic materials.

**S11 Infrared Spectroscopy**

All the gels were prepared in vials by applying the method described in Section 3. The gels were dried in vacuum prior to analysis. The xerogels were placed into the infrared spectrophotometer and the IR spectra were recorded from 4000 cm^-1^ to 450 cm^-1^. We used FT-IR methods to see whether we could observe different environments for C=O and O-H/N-H stretches between the individual gels and the multi-component system. However, it was not possible to make this distinction, with the IR peaks for the multi-component system appearing as a superposition of the individual gel infra-red spectra.


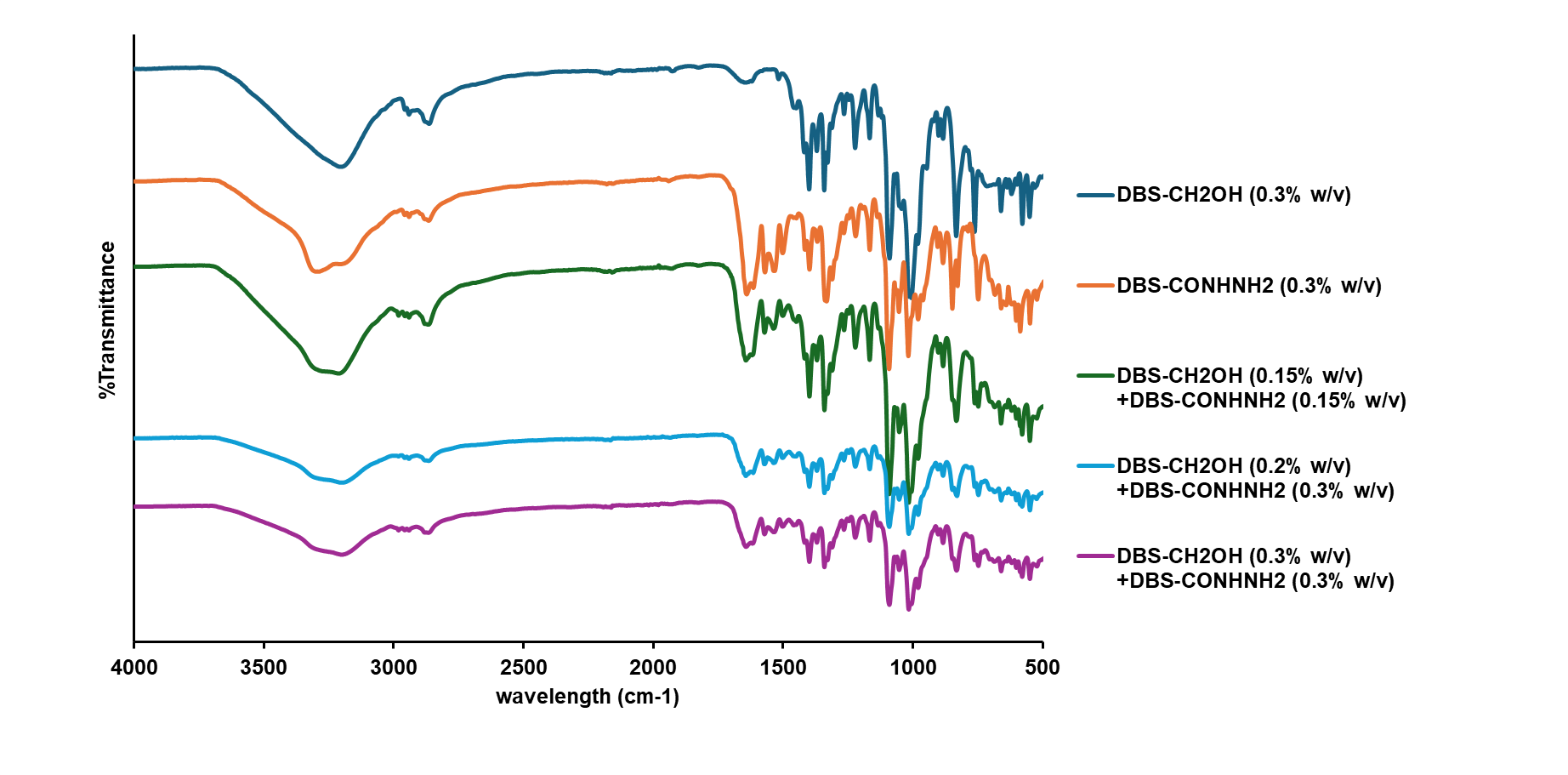
Figure S24. IR spectra of xerogels obtained from DBS-CH_2_OH, DBS-CONHNH_2_, and DBS-CH_2_OH/DBS-CONHNH_2_ with different loadings of DBS-CH_2_OH:DBS-CONHNH_2_.

**S12 Biological studies**

**S12.1 Viability Tests.** A hot solution of DBS-CH_2_OH (0.2 or 0.3% w/vol), DBS-CONHNH_2_ (0.3% wt/vol), or DBS-CH_2_OH/DBS-CONHNH_2_ (0.2 or 0.3% wt/vol DBS-CH_2_OH combined with 0.3% wt/vol DBS-CONHNH_2_) was prepared in autoclaved water and then 75 μL of the relevant sample was transferred to each well of non-treated 96-well plates. After 24 h, the gels were then washed with DMEM (200 μL, 10% FBS, 1% P/S) six times every 30-45 min. After gel washing, the gels were soaked with 100 μL of DMEM (10% FBS, 1% P/S) and Y201 MSCs (25,000 cells/well) were seeded. Finally, DMEM (10% FBS, 1% P/S) was added to reach 200 μL of solution per well. A control experiment was performed by soaking the gel in DMEM 200 μL without cells for background subtraction. Afterwards, the systems were cultured at 37 °C without exchanging fresh medium. Cell viability was measured at day 0, 3, 6 and 9. The spent medium was removed from each well and Alamar Blue solution (100 μL, 10% in DMEM) was added. The plate was incubated at 37 °C for 4 hours. After that, aliquots (40 μL) were taken from each well and then transferred to each well of a new 96-well plate containing 160 μL of DMEM (10% FBS, 1% P/S). Control experiments without cells were performed for each gel. This experiment was performed in sextuplicate. Fluorescence signals were measured using a fluorescence plate reader with an excitation wavelength at 530-560 nm and an emission wavelength of 590 nm. The fluorescence of the gel alone (control) was subtracted from the fluorescence signal of cell culture on gel. Statistical analysis was performed using Graphpad Prism version 10 for Window. A 2-way ANOVA was performed, and outcomes are displayed as mean +/- SEM with significant differences (**p<0.0001, ***p<0.001, **p<0.01, *p<0.05).

**S12.2 Live-Dead Staining.** 400 μL of DBS-CH_2_OH (0.2 or 0.3% wt/vol), DBS-CONHNH_2_ (0.3% wt/vol) and DBS-CH_2_OH/ DBS-CONHNH_2_ (0.2 or 0.3% wt/vol for DBS-CH_2_OH and 0.3% wt/vol for DBS-CONHNH_2_) were prepared in non-treated 24 well plates using autoclaved water. Live-Dead staining: 50 μL of anhydrous DMSO was added into Calcein AM (C3100, 50 μg), yielding a 1 mM stock solution. The Calcein AM stock solution (20 μL, 1 mM) and propidium iodide (20 μL, 1 mg/mL) were mixed and diluted with phenol red free media (10 mL in total volume, 10% FBS, 1% P/S) to a final working solution (2 μM of calcein AM and 2 μg/mL of propidium iodide). At different time points, spent media were removed, and the working solution (0.5 mL) was gently added to the gel samples. The samples were incubated at 37 °C for 15 min. After this time, the samples were washed with fresh media (0.6 mL) twice for every 5 min at 37 °C. The images of different gels were collected using and a Zeiss LSM 880 with Airyscan confocal microscopes (Figs. S25-S29).

Figure S25. 3D confocal microscopy images (top) and z-axis maximum projection images (below) of Y201 MSCs growth on DBS-CH_2_OH (0.2% wt/vol) showing live stain (green) and dead stain (red) at day 0, 3 and 6. Scale bar of 100 μm.


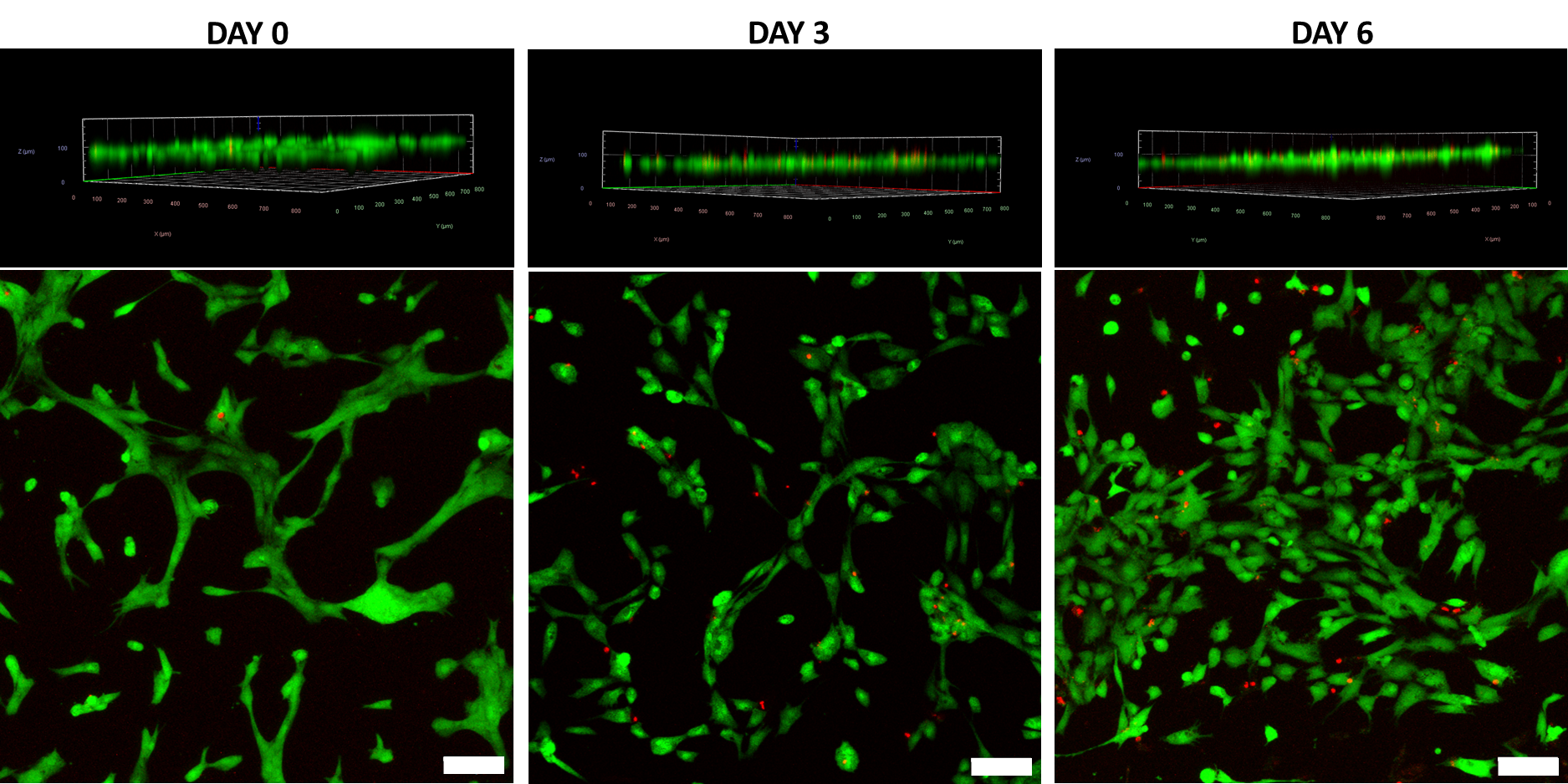

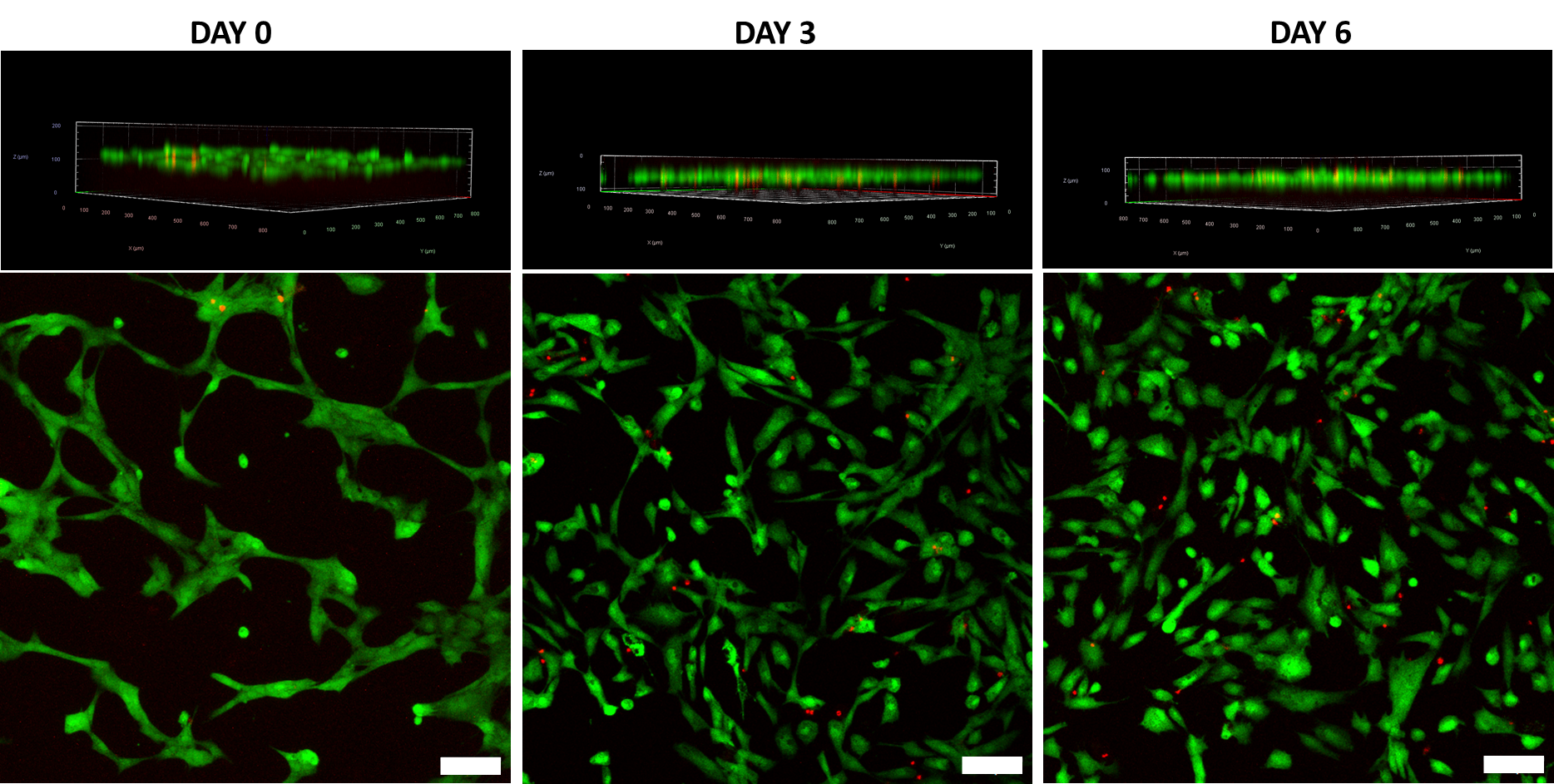


Figure S26. 3D confocal microscopy images (top) and z-axis maximum projection images (below) of Y201 MSCs growth on DBS-CH_2_OH (0.3% wt/vol) showing live stain (green) and dead stain (red) at day 0, 3 and 6. Scale bar of 100 μm.


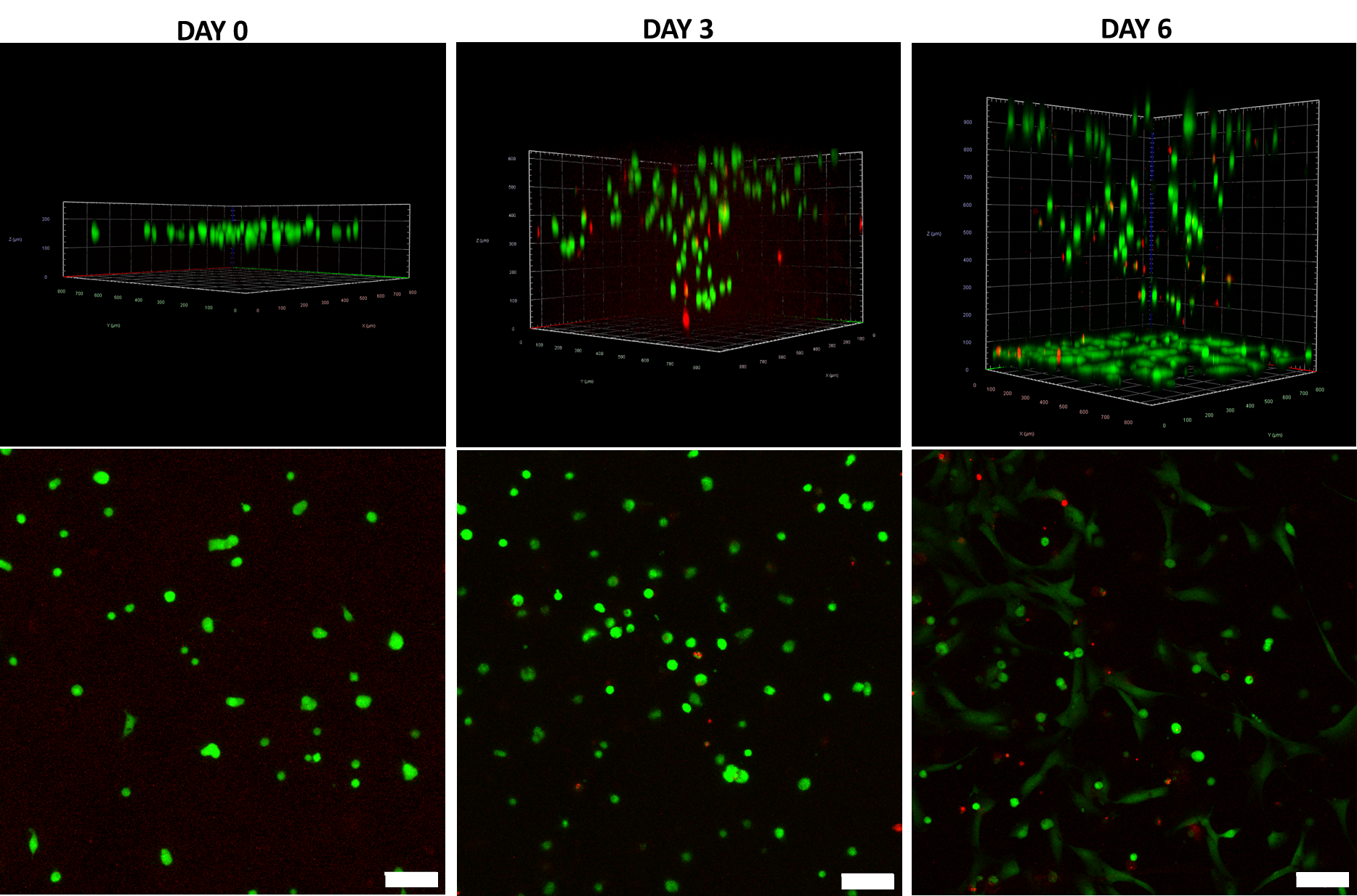


Figure S27. 3D confocal microscopy images (top) and z-axis maximum projection images (below) of Y201 MSCs growth on DBS-CONHNH_2_ (0.3% wt/vol) showing live stain (green) and dead stain (red) at day 0, 3 and 6. Scale bar of 100 μm.

Figure S28. 3D confocal microscopy images (top) and z-axis maximum projection images (below) of Y201 MSCs growth on DBS-CH_2_OH/DBS-CONHNH_2_ (0.2% wt/vol for DBS-CH_2_OH and 0.3% wt/vol for DBS-CONHNH_2_) showing live stain (green) and dead stain (red) at day 0, 3 and 6. Scale bar of 100 μm.


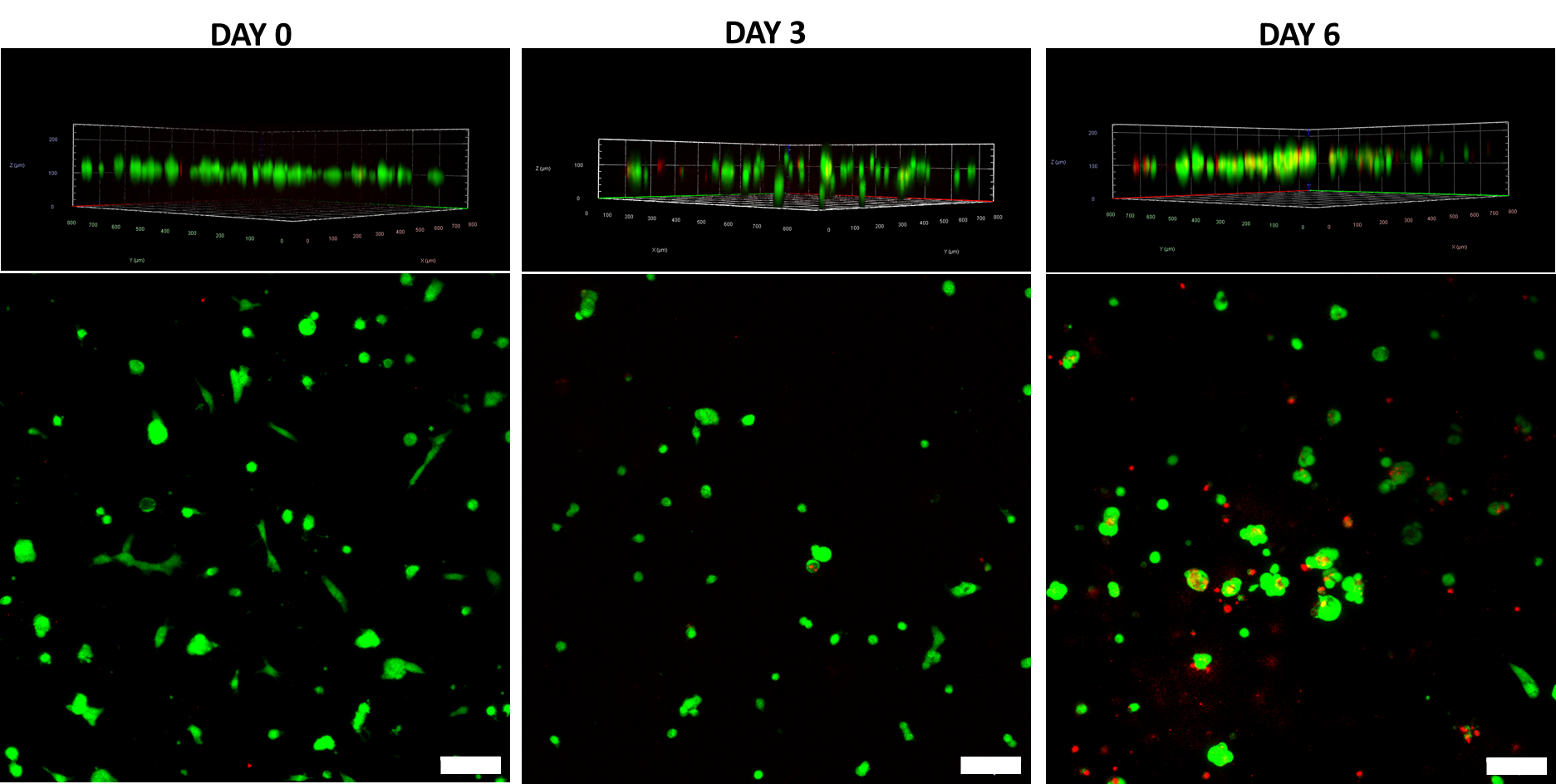

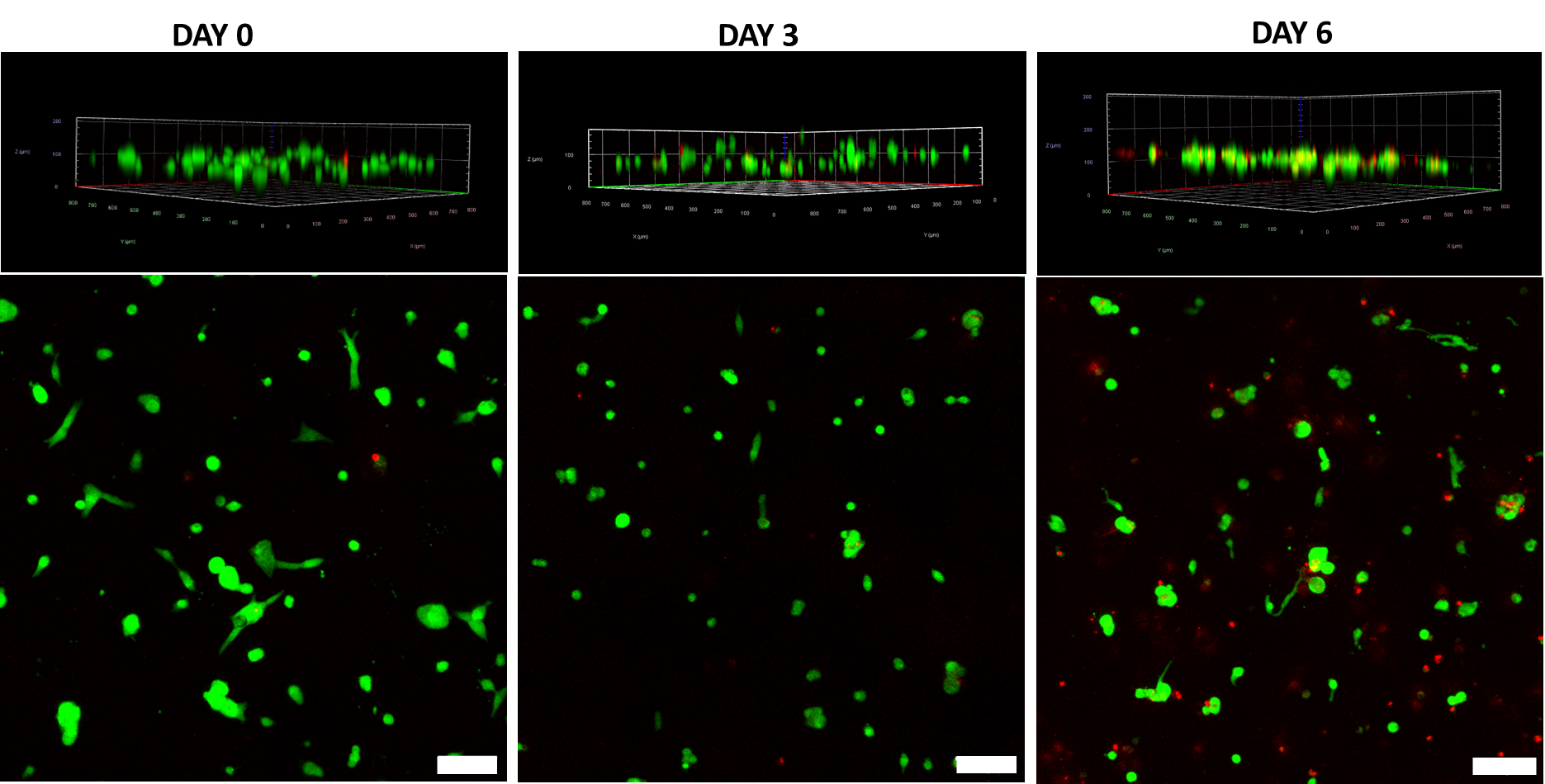


Figure S29. 3D confocal microscopy images (top) and z-axis maximum projection images (below) of Y201 MSCs growth on DBS-CH_2_OH/DBS-CONHNH_2_ (0.3% wt/vol for each gelator) showing live stain (green) and dead stain (red) at day 0, 3 and 6. Scale bar of 100 μm.

**S12.3 Positive stain control experiment with propidium iodide on hydrogels.** This experiment was performed to confirm that propidium iodide stained dead cells, showing the red colour. Hydrogels (0.4 mL) were prepared in each well of 24 well plate as described above in Section 12.2. The cells (25,000 cells) were seeded into each well and covered with phenol red free media (0.7 mL, 10% FBS, 1% P/S). Over 24 h, spent media were removed, and samples were washed with DPBS (1x, 0.5mL) twice. Paraformaldehyde (PFA) in DPBS (4% v/v, 0.4 mL) was added to fix the cells at 4 °C overnight. Next day, PFA was removed, washed with DPBS (1x, 0.5mL) twice. The working solution of propidium iodide (0.5 mL, 1 μg/mL) prepared in the media was added to each well, followed by incubation at room temperature for 15 min. The samples were washed with fresh media (0.5 mL) twice. The images (Fig. S30) were collected using a Zeiss AXIO Observer 7 inverted Fluorescence motorized XYZ Definite Focus Microscope.


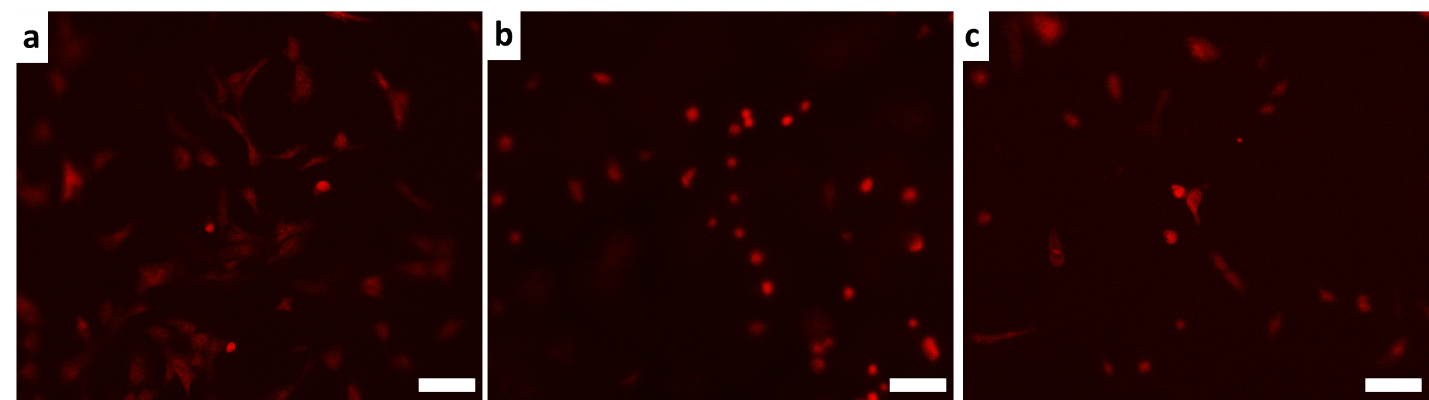


Figure S30. Fluorescence images of Y201 MSCs growth on (a) DBS-CH_2_OH (b) DBS-CONHNH_2_ and (c) DBS-CH_2_OH/DBS-CONHNH_2_ after fixing and staining with propidium iodide. Scale bar 100 μm.

**S12.4 Live-Dead Cell Counting.** Images collected via the live-dead cell staining methodology described in Section 12.2 above were quantitatively analysed to count live and dead cells. For each gel, 3-5 images were analysed at each of Day 0, 3 and 6. Two researchers then independently analysed these images with the assistance of software packages such as ImageJ. Their individual resulting cell counts were averaged to give a mean count of live and dead cells for each image. In total 37 images were analysed in duplicate to create the data illustrated in Figure S30.


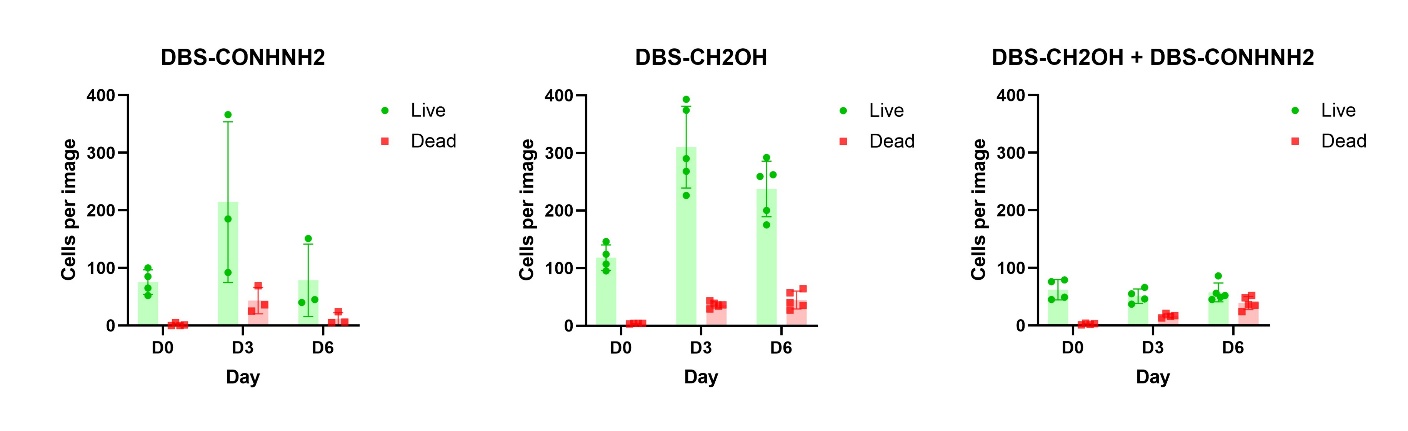
Figure S31. Live-dead cell count plots at Days 0, 3 and 6 for gels based on DBS-CONHNH_2_ (0.3% wt/vol), DBS-CH_2_OH (0.3% wt/vol) and DBS-CH_2_OH + DBS-CONHNH_2_ (each 0.3% wt/vol).

**S13 References**

1. D. J. Cornwell, B. O. Okesola, D. K. Smith, *Soft Matter*, **2013**, *9*, 8730-8736.

2. P. R. A. Chivers, J. A. Kelly, M. J. S. Hill, D. K. Smith, *React. Chem. Eng.*, **2020**, *5*, 1112-1117.
